# Distributed non-disclosive validation of predictive models by a modified ROC-GLM

**DOI:** 10.1186/s12874-024-02312-4

**Published:** 2024-08-29

**Authors:** Daniel Schalk, Raphael Rehms, Verena S. Hoffmann, Bernd Bischl, Ulrich Mansmann

**Affiliations:** 1 Department of Statistics, LMU Munich, Munich, Germany; 2 Institute for Medical Information Processing, Biometry and Epidemiology, LMU Munich, Munich, Germany; 3 DIFUTURE (DataIntegration for Future Medicine, www.difuture.de), LMU Munich, Munich, Germany; 4 Munich Center for Machine Learning (MCML), LMU Munich, Munich, Germany

**Keywords:** Area under the ROC curve, Distributed computing, Medical tests, ROC-GLM

## Abstract

**Background:**

Distributed statistical analyses provide a promising approach for privacy protection when analyzing data distributed over several databases. Instead of directly operating on data, the analyst receives anonymous summary statistics, which are combined into an aggregated result. Further, in discrimination model (prognosis, diagnosis, etc.) development, it is key to evaluate a trained model w.r.t. to its prognostic or predictive performance on new independent data. For binary classification, quantifying discrimination uses the receiver operating characteristics (ROC) and its area under the curve (AUC) as aggregation measure. We are interested to calculate both as well as basic indicators of calibration-in-the-large for a binary classification task using a distributed and privacy-preserving approach.

**Methods:**

We employ DataSHIELD as the technology to carry out distributed analyses, and we use a newly developed algorithm to validate the prediction score by conducting distributed and privacy-preserving ROC analysis. Calibration curves are constructed from mean values over sites. The determination of ROC and its AUC is based on a generalized linear model (GLM) approximation of the true ROC curve, the ROC-GLM, as well as on ideas of differential privacy (DP). DP adds noise (quantified by the $\ell _2$ sensitivity $\Delta _2(\hat{f})$) to the data and enables a global handling of placement numbers. The impact of DP parameters was studied by simulations.

**Results:**

In our simulation scenario, the true and distributed AUC measures differ by $\Delta \text {AUC} < 0.01$ depending heavily on the choice of the differential privacy parameters. It is recommended to check the accuracy of the distributed AUC estimator in specific simulation scenarios along with a reasonable choice of DP parameters. Here, the accuracy of the distributed AUC estimator may be impaired by too much artificial noise added from DP.

**Conclusions:**

The applicability of our algorithms depends on the $\ell _2$ sensitivity $\Delta _2(\hat{f})$ of the underlying statistical/predictive model. The simulations carried out have shown that the approximation error is acceptable for the majority of simulated cases. For models with high $\Delta _2(\hat{f})$, the privacy parameters must be set accordingly higher to ensure sufficient privacy protection, which affects the approximation error. This work shows that complex measures, as the AUC, are applicable for validation in distributed setups while preserving an individual’s privacy.

**Supplementary Information:**

The online version contains supplementary material available at 10.1186/s12874-024-02312-4.

## Introduction

Medical research needs trust that the use of confidential patient data follows principles of privacy protection. However, depending on the released data, breaches of the patient’s privacy may occur [16]. Even when a patient gives informed consent that the researcher can have access to his/her pseudonymized patient data, it is necessary to keep data in a protected environment and to process it accordingly. Privacy-preserving modeling protects sensitive patient data [1].

Typically, multi-center studies in medicine or epidemiology collect the data in a central study database and perform the analyses in a specifically protected environment following the informed consent of the study subjects. However, this requires an administratively challenging and time-consuming trustworthy data-sharing process.

Using only anonymous and aggregated data for analysis can alleviate the administrative load for data sharing. Distributed data networks in clinical studies allow to leverage routinely collected electronic health data and thus streamline data collection. Non-disclosing distributed analysis is an important part of this concept. It enables statistical analyses without sharing individual patient data (IPD) between the various sites of a clinical study or sharing IPD with a central analysis unit. Non-disclosing distributed analyses protect patient data privacy and enhance data security, making this a potentially advantageous approach for medical research involving sensitive patient data. However, algorithms are needed to support robust multivariable-adjusted statistical analysis without the need to centralize IPD.

As a part of the German Medical Informatics Initiative^1^ (MII) the Data Integration for Future Medicine (DIFUTURE) consortium [21] undertakes distributed data network studies and provides tools as well as algorithms for non-disclosing distributed analyses. DIFUTURE’s specific objective is to provide digital tools for individual treatment decisions and prognosis and to develop distributed algorithms for the discovery and validation of prognostic and predictive rules. In the following paper, we investigate how the area under the curve (AUC) and its confidence intervals (CIs) proposed by DeLong et al. [6] behave if the computed AUC uses a generalized linear model (GLM) approach of Pepe [19] in a distributed differential privacy framework. We can also determine and view the ROC using distributed analyses.

The concept of differential privacy was operationalized by Dwork [7]. An algorithm is considered to be differential private if an observer cannot determine based solely on the output whether a particular individual’s information was used in the computation. Differential privacy ensures protection of patient data privacy, as differential private algorithms are more likely to resist identification and re-identification attacks [8] than alternative approaches.

The ROC curve and its AUC in pooled IPD testing data as well as assessing the quality of calibration [27] is the state-of-the-art of prognostic/predictive validation techniques in a binary classification setting. In general, IPD transfer requires specific patient consent, and data protection laws apply. Here, we present a non-disclosing distributed ROC-GLM, which we use to calculate the ROC curve, its AUC, and the respective CIs. These methods and their implementation in DataSHIELD framework [10] allow analyses in which IPD does not leave its secured environment. This way, only noisy IPD under differential privacy or anonymous and aggregated statistics are shared, thereby preventing the identification of individuals. We also demonstrate that assessing the calibration of binary classification rules based on distributed calculation is a straightforward task.

We motivate our approach by looking at the binormal classification case, where individuals with negative or positive outcome have $\mathcal {N}(\mu _0, \sigma _0^2)$ or $\mathcal {N}(\mu _1, \sigma _1^2)$ distributed scores with $\mu _0 < \mu _1$. With $a = (\mu _1 - \mu _0)/\sigma _1$ and $b = \sigma _0 / \sigma _1$ it holds that $\text {ROC}(t) = \Phi (a+b\cdot \Phi ^{-1}(t))$ and $AUC = \Phi (a/(1+b^2)^{0.5})$. In the case of non-normal score distribution, the ROC-GLM allows to approximate the respective ROC and AUC by using the same expressions where *a* and *b* are estimated from a probit regression. Furthermore, the ROC-GLM approach allows a simultaneous estimation of ROC curves and AUCs over a set of subgroups defined by covariates [19].^2^

### Contribution

The work herein proposes new privacy-preserving algorithms adapted to the distributed data setting for the ROC-GLM [18], the AUC derived therefrom, and its CIs for that AUC. To validate the algorithms, we provide a simulation study to assess estimation accuracy. We compare the results with those from the standard procedure. Furthermore, we apply the proposed algorithms to validate a given prognostic rule on data of breast cancer patients.

We describe how the concept of the distributed ROC analysis can be incorporated into the ROC-GLM by using differential privacy. We generate privacy-preserving survivor function that can be communicated without the threat of privacy breaches. Furthermore, we outline a distributed Fisher scoring algorithm [14] that estimates parameters for the ROC-GLM. In addition, we describe a privacy protecting distributed calibration approach and demonstrate that distributed GLM model building does not impose specific algorithmic challenges. Furthermore, we introduce a distributed version of the Brier score [5] and the calibration curve [28]. The bycatch of the principles described is a privacy protected version of the binormal ROC and its AUC.

## Related literature

Boyd et al. [3] calculate the AUC under differential privacy using a symmetric binormal ROC function. However, our approach is more general and allows extension to non-parametric data with multiple covariates. While they derive the AUC from the ROC parameters, we also use integration techniques. In addition, we provide CIs for the AUC. Ünan et al. [25] use homomorphic encryption to calculate the ROC curve. Their approach does not provide CIs or an extension to multiple covariates. To the best of our knowledge, a modified ROC-GLM algorithm for non-disclosing distributed analyses has so far not been developed.

## Background

Throughout this paper, we consider binary classification, with 1 for a case with the trait(s) of interest (i.e., “diseased”, “success”, “favorable”) and 0 for the remaining cases (i.e., lacking trait(s) of interest, “healthy”, “no success”, “unfavorable”). Furthermore, $f(\varvec{x})\in \mathbb {R}$ is the true score based on a true but unknown function *f* for a patient with a feature vector $\varvec{x}$ (the individual realization of an underlying random vector $\varvec{X}$). In this paper, the score can also express a posterior probability with $f(\varvec{x}) \in [0,1]$. The function *f* is estimated by a statistical (classification) model ${\hat{f}}:\mathbb {R}^{p} \rightarrow \mathbb {R}$. The estimated individual score for a subject with feature or covariate vector $\varvec{x}\in \mathbb {R}^{p}$ is ${\hat{f}}(\varvec{x})$. The training or validation data set used to fit or validate ${\hat{f}}$ is denoted as $\mathcal {D} = \{(\varvec{x}_1, y_1), \dots , (\varvec{x}_n, y_n) \}$ with $y_i \in \{1, {0}\}$. The score ${\hat{f}}(\varvec{x})$ and a threshold value $c\in \mathbb {R}$ are used to define a binary classifier: $\mathbb {1}_{[c,\infty )}({{\hat{f}}(\varvec{x})})$. On an observational level, $\varvec{x}_{1,i}$ and $\varvec{x}_{{0},i}$ indicate the $i^{\text {th}}$ observation that corresponds to a positive or negative output *y*. The number of observations in $\mathcal {D}$ with output 1 and 0 are denoted by ${n_{1}}$ and ${n_{0}}$. The set of scores that corresponds to the positive or negative output is denoted by $\mathcal {F}_{1} = \{{\hat{f}}(\varvec{x}_{1,i})\ |\ i = 1, \dots , {n_{1}}\}$ and $\mathcal {F}_{0} = \{{\hat{f}}(\varvec{x}_{{0},i})\ |\ i = 1, \dots , {n_{0}}\}$, with $\mathcal {F}_{1, i} = {\hat{f}}(\varvec{x}_{1,i})$ and $\mathcal {F}_{{0}, i} = {\hat{f}}(\varvec{x}_{{0},i})$.

### ROC curve and AUC

To quantify the quality of a binary classifier, we use the true positive rate (TPR) and false positive rate (FPR) with values between 0 and 1: $\textsf{TPR}(c) = P(f(\varvec{X}) \ge c\ |\ Y = 1)$ and $\textsf{FPR}(c) = P(f(\varvec{X}) \ge c\ |\ Y = 0)$ for threshold $c\in \mathbb {R}$ [18]. These probability functions are also known as positive or negative * survivor functions* $S_{1}(c) = \textsf{TPR}(c)$ and $S_{0}(c) = \textsf{FPR}(c)$. The ROC curve is defined as $\text {ROC}(t) = S_{1}(S_{0}^{-1}(t))$. The AUC as a measure of discrimination between the two distributions of the positive and negative class is given as $AUC = \int _0^1 \text {ROC}(t)\ dt$ [30].

### Empirical calculation of the ROC curve and AUC

The calculation of the empirical ROC curve uses the *empirical survivor functions* $\hat{S}_{1}$ and $\hat{S}_{0}$. These functions are based on the empirical cumulative distribution functions (ECDF) $\hat{F}_{1}$ of $\mathcal {F}_{1}$ and $\hat{F}_{0}$ of $\mathcal {F}_{0}$: $\hat{S}_{1} = 1 - \hat{F}_{1}$ and $\hat{S}_{0} = 1 - \hat{F}_{0}$. The set of possible values of the empirical TPR and FPR are given by $\mathcal {S}_{1} = \{\hat{S}_{1}({\hat{f}}(\varvec{x}_{{0},i}))\ |\ i = 1, \dots , {n_{0}}\}$ and $\mathcal {S}_{0} = \{\hat{S}_{0}({\hat{f}}(\varvec{x}_{1,i}))\ |\ i = 1, \dots , {n_{1}}\}$ and are also called *placement values*. These values standardize a given score relative to the class distribution [19]. The set $\mathcal {S}_{1}$ represents the positive placement values and $\mathcal {S}_{0}$ the negative placement values.

The empirical version of the $\text {ROC}(t)$ is a discrete function derived from the placement values $\mathcal {S}_{1}\subseteq \{0, 1/{n_{1}}, \dots , ({n_{1}}-1)/{n_{1}}, 1\}$ and $\mathcal {S}_{0}\subseteq \{0, 1/{n_{0}}, \dots , ({n_{0}}-1)/{n_{0}}, 1\}$. The empirical AUC is a sum over rectangles of width $1 / {n_{0}}$ and height $\hat{S}_{1}({\hat{f}}(\varvec{x}_{{0},i}))$ ([19], p.106):

1 $$\begin{aligned} \widehat{AUC} = {n_{0}}^{-1}\sum \limits _{i=1}^{{n_{0}}} \hat{S}_{1}({\hat{f}}(\varvec{x}_{{0},i})). \end{aligned}$$

Equation (1) is the empirical analogue of the expectations of the placement values, i.e. $AUC = E(S_{1}(f(\varvec{x})))$. The term ${\hat{f}}(\varvec{x}_{{0},i})$ is the score of the estimated statistical model for the negative output $\varvec{x}_{{0},i}$. The empirical AUC is a function of the empirical survivor function $\hat{S}_{1}$ evaluated at the score values for all negative outputs $\varvec{x}_{{0},i}$.

The empirical AUC is equivalent to the Mann-Whitney U-statistic and inherits the respective distributional properties. For a sufficient large sample of $n_0$ and $n_1$, it converges to the normal distribution.

### CI for the empirical AUC

CIs are calculated following [6]. The variance of the empirical AUC is determined by:

2 $$\begin{aligned} \widehat{\textsf{var}}(AUC) = \frac{\widehat{\textsf{var}}(\mathcal {S}_{1})}{{n_{0}}} + \frac{\widehat{\textsf{var}}(\mathcal {S}_{0})}{{n_{1}}}. \end{aligned}$$

An asymmetric confidence interval which guarantees values within the interval (0,1) is derived from a symmetric confidence interval for logit AUC ${\textsf{ci}}_\alpha \left( \textsf{logit}({AUC})\right)$ using the $\textsf{logit}^{-1}$ transformation (p 107, [19]):

3 $$\begin{aligned} {\textsf{ci}}_\alpha \left( \textsf{logit}({AUC})\right) = \textsf{logit}({\widehat{AUC}}) \pm \Phi ^{-1}\left( 1 - \frac{\alpha }{2}\right) \frac{\sqrt{\widehat{\textsf{var}}\left( AUC\right) }}{\widehat{AUC}\left( 1 - \widehat{AUC}\right) }. \end{aligned}$$

The term $\Phi ^{-1}$ denotes the quantile function of the standard normal distribution. The term of the standard error is a direct consequence of the application of the delta rule to logit(AUC).

Statistical testing can be conducted based on that CI. For example, the hypothesis $H_0: AUC \le a_0$ vs. $H_1: AUC > a_0$ with a significance level of $\alpha$ can be tested by checking whether $\textsf{logit}({a_0}) < a$, $\forall a \in \textsf{ci}_{\alpha }$ to reject $H_0$.

### The ROC-GLM

The ROC-GLM interprets the ROC curve as a GLM ([19], Section 5.5.2): $\text {ROC}_{g}(t | \varvec{\gamma }) = g(\varvec{\gamma } h(t))$, with link function $g:\mathbb {R}\rightarrow [0,1], \eta \mapsto g(\eta )$, coefficient vector $\varvec{\gamma }\in \mathbb {R}^l$, and covariate vector $h: \mathbb {R}\rightarrow \mathbb {R}^l, t \mapsto \varvec{h}(t) = (h_1(t), \dots , h_l(t))^{\textsf{T}}$. In general this estimator is not unbiased (see for example Appendix A.6).

Estimating the ROC-GLM uses an intermediate data set $\mathcal {D}_{\text {ROC-GLM}} = \{(u_{ij}, \varvec{h}(t_j))\ | \ i = 1, \dots , {n_{1}}, j = 1, \dots , n_T\}$ with covariates $\varvec{h}(t_j)$, a set of thresholds $T = \{t_1, \dots , t_{n_T}\}$, and binary response $u_{ij}\in \{0,1\}$, $u_{ij} = \mathbb {1}_{(\hat{S}_{0}(\mathcal {F}_{1, i}),\infty )}({t_j}) = \mathbb {1}_{(-\infty ,\mathcal {F}_{1, i}]}({\hat{S}_{0}^{-1}(t_j)})$. The simplest ROC-GLM uses the two-dimensional vector $\varvec{h}(t)$ with $h_1(t) = 1$ and $h_2(t) = \Phi ^{-1}(t)$. Setting the link function to $g = \Phi$ results in the binormal form $\text {ROC}_{g}(t|\varvec{\gamma }) = \Phi (\gamma _1 + \gamma _2\Phi ^{-1}(t))$. It is equivalent to a probit regression with response variable $u_{ij}$ and covariate $\Phi ^{-1}(t_j)$. A common strategy for choosing the set of thresholds *T* is to use an equidistant grid.

**Fig. 1:**
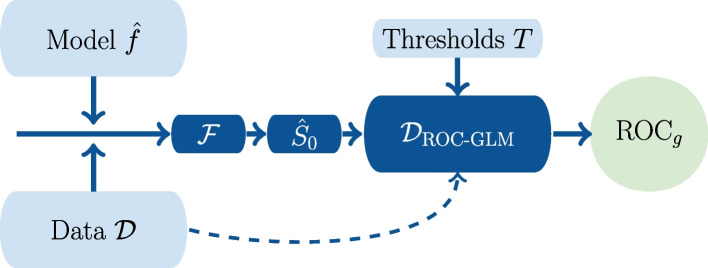
The $\text {ROC-GLM}(\mathcal {D})$ procedure starts with the data $(\mathcal {D})$ and a model *f* for predicting scores *Y*. It calculates the survivor function $\hat{S}_{\bar{D}}$ and determines the intermediate data $\mathcal {D}_{\text {ROC-GLM}}$. The probit regression estimates the parameters

The estimated ROC curve $\text {ROC}_{g}(t | \hat{\varvec{\gamma }})$ results from the estimated model parameters $\hat{\varvec{\gamma }}$. The AUC from the ROC-GLM $\widehat{AUC}_{\text {ROC-GLM}}$ is the integral $\widehat{AUC}_{\text {ROC-GLM}} = \int _0^1 \text {ROC}_{g}(t|\hat{\varvec{\gamma }})\ dt$. Here, we use the R-function integrate [20] or the explicit formula $AUC = \Phi (a/(1+b^2)^{0.5})$. Figure 1 visualizes the single steps of the ROC-GLM algorithm.

### Differential privacy

Differential privacy (DP) is a theoretical framework which provides formal guarantees to restrict privacy leakage of individual information when statistical analysis is performed on the data [9, 26]. One of the most prominent DP approaches adds noise $\varvec{r}$ to a deterministic algorithm to obtain a randomized version $\mathcal {M}: \mathcal {X} \mapsto \mathcal {Y}$ with domain $\mathcal {X}$ (e.g., $\mathcal {X} = \mathbb {R}^p$) and target domain $\mathcal {Y}$ (e.g., $\mathcal {Y} = \mathbb {R}$ in regression). Formally speaking a mechanism $\mathcal {M}$ is $(\varepsilon , \delta )$-differential private, if for any subset of outputs $R \subseteq \mathcal {Y}$, the property $P(\mathcal {M}(\varvec{x}) \in R) \le \exp (\varepsilon ) P(\mathcal {M}(\varvec{x}^\prime ) \in R) + \delta$ holds for two adjacent inputs.^3^ The value of $\varepsilon$ controls how much privacy is guaranteed. Intuitively, this means that for a small $\varepsilon$, applying the randomized algorithm $\mathcal {M}$ on two datasets that only differ in one data point, the typical output (i.e. a high probability) of $\mathcal {M}$ for both datasets has to be nearly the same while a larger value of $\varepsilon$ would allow that the typical output could differ more. The value of $\delta$ can be interpreted as the probability that $\varepsilon$-differential privacy is broken (see [8]). Hence, $\delta$ has to be set to a small value that should be at least less than the inverse number of data points. We provide an interpretation of the privacy parameter $\varepsilon$ in Appendix A.3.

We add normally distributed noise $\varvec{r}$ to ${\hat{f}}$ to obtain a private version of the estimated scores ${\hat{f}}(\varvec{x})$ (i.e. *Gaussian mechanism*): $\mathcal {M}(\varvec{x}) = {\hat{f}}(\varvec{x}) + r$. Hence, the obfuscated values of the survivor function $\tilde{\mathcal {F}}_{1} = \{\mathcal {M}(\varvec{x}_{1,i})\ |\ i = 1, \dots , {n_{1}}\}$ and not the original score values $\mathcal {F}_{1}$ are used for further calculations. The noise $\varvec{r}$ follows a zero-mean Gaussian $\mathcal {N}(0, \tau ^2)$, where its variance is set to the minimal value that guarantees a certain level of privacy. Balle and Wang [2] propose the *analytic Gaussian mechanism* which searches numerically for a minimal value of $\tau$ such that a defined level of privacy ($\varepsilon$, $\delta$) for a given $\ell _2$-sensitivity is achieved. The sensitivity of an algorithm is defined as $\Delta _2(\hat{f}) = \sup _{\text {adjacent}\ \varvec{x},\ \varvec{x}^\prime } \Vert {\hat{f}}(\varvec{x}) - {\hat{f}}(\varvec{x}^\prime )\Vert _2$. Within this work, we first calculate the $\ell _2$-sensitivity of the prediction model $\hat{f}$ to determine possible values of the privacy parameters (see Correctness of the AUC inferred from ROC-GLM and distributed ROC-GLM section). Given these parameters, we subsequently determine the minimal required amount of noise $\tau$ for the analytic Gaussian mechanism. We provide further details and a visualization of the Gaussian mechanism in Appendix A.2.

## Distributed ROC-GLM

### General principles

A total of *K* data sets are distributed over a network of *K* sites: $\mathcal {D}^{(1)}, \dots , \mathcal {D}^{(K)}$. Each data set $\mathcal {D}^{(k)}$ consists of $n^{(k)}$ observations $(\varvec{x}_i^{(k)}, y_i^{(k)})$. The $j^{\text {th}}$ component of the $i^{\text {th}}$ feature vector of the $k^{\text {th}}$ site is denoted by $x_{j,i}^{(k)}$. The $i^{\text {th}}$ outcome on site *k* is $y_i^{(k)}$. We assume (1) the single data have empty intersections and (2) the union of the distributed data is a subset of the full but inaccessible data set:

4 $$\begin{aligned} \mathcal {D} =\bigcup _{k=1}^K \mathcal {D}^{(k)},\ \ n = n^{(1)} + \dots + n^{(K)} \end{aligned}$$

Instead of calculating the ROC-GLM for one local data set, we want to calculate the ROC-GLM on *K* distributed data sets $\mathcal {D}^{(1)}, \dots , \mathcal {D}^{(K)}$. All shared information must comply with the following non-disclosing principles:

**A1**: Given the value *q*, the *privacy level*, an aggregation $a: \mathbb {R}^d \mapsto \mathbb {R}$, $\varvec{v} \rightarrow a(\varvec{v})$ is admissible for sharing the value $a(\varvec{v})$ if $d \ge q \in \mathbb {N}$. The *privacy level* requests a minimum number of values on which $a(\varvec{v})$ is derived. In the distributed setup, the aggregation $a(\varvec{v}^{(k)})$ with $n^{(k)}$ unique values in $\varvec{v}^{(k)}$ shared from each of the *K* sites can then be further processed. Values $a(\varvec{v}^{(k)})$ can be shared if $n^{(k)} \ge q$.
**A2**: Differential privacy [7] is used to ensure non-disclosive IPD via a noisy representation.

#### Distributed Brier score and calibration curve

Calibration of a probabilistic (or scoring) classifier is often addressed by the Brier score [5] or a calibration curve [28]. Both can be calculated by considering criterion ${\textbf {A1}}$.

*Brier score:* The Brier score ($\text {BS}$) is the mean squared error of the true 0-1-labels and the predicted probabilities of belonging to class 1. For the Brier score, the score ${\hat{f}}(\varvec{x}) \in [0,1]$ is given as posterior probability. The Brier score is calculated by:

5 $$\begin{aligned} \text {BS} = n^{-1}\sum \limits _{i=1}^n \left( y_i - {\hat{f}}(\varvec{x}_i)\right) ^2 \end{aligned}$$

Hence, having a prediction model $\hat{f}$ at each of the *K* sites, we can calculate the Brier score by:

1. Calculating the residuals $e_i^{(k)}$ based on the true label $y_i^{(k)}$ at site *k* and the predicted probabilities $\hat{f}(\varvec{x}_i^{(k)})$: $e_i^{(k)} = y_i^{(k)} - \hat{f}(\varvec{x}_i^{(k)})$, $\forall i = 1, \dots , n^{(k)}$.
2. Calculating $a_{\text {sum}}(\varvec{e}^{(k)} \circ \varvec{e}^{(k)})$, with $\varvec{e}^{(k)} = (e_1^{(k)}, \dots , e_{n^{(k)}}^{(k)})^{\textsf{T}}\in \mathbb {R}^{n_k}$, the element-wise product $\circ$, and aggregation $a_{\text {sum}}(\varvec{v}^{(k)}) = \sum \nolimits _{i=1}^{n^{(k)}} v_i^{(k)}$.
3. Sending $a_{\text {sum}}(\varvec{e}^{(k)} \circ \varvec{e}^{(k)})$ and $n^{(k)}$ (if $n_k \ge q$) to the host, who finally calculates $\text {BS} = n^{-1}\sum \nolimits _{k=1}^K a_{\text {sum}}(\varvec{e}^{(k)} \circ \varvec{e}^{(k)})$.

*Calibration curve:* To calculate a calibration curve, we discretize the domain of the probabilistic classifier $$\hat{f}$$ in [0, 1] into $$n_{\text {bin}}$$ bins (for example, $$n_{\text {bin}} + 1$$ equidistant points $$p_i$$ from 0 to 1 to construct the $$n_{\text {bin}}$$ bins $$b_l = [p_l, p_{l+1})$$ for $$l = 1, \dots , n_{\text {bin}} - 1$$ and $$b_{n_{\text {bin}}} = [p_{n_{\text {bin}}}, p_{n_{\text {bin}} + 1}]$$ for $$l = n_{\text {bin}}$$). The calibration curve is the set of 2-dimensional points $$p_{\text {cal},l} = (\text {pf}_l, \text {tf}_l)$$, with $$\text {tf}_l = |\mathcal {I}_l|^{-1}\sum \nolimits _{i \in \mathcal {I}_l} y_i$$ as the true fraction of $$y_i = 1$$ in bin $$b_l$$ and $$\text {pf}_l = |\mathcal {I}_l|^{-1}\sum \nolimits _{i \mathcal {I}_l} \hat{f}(\varvec{x}_j)$$ as the predicted fraction for outcome 1 in $$b_l$$. The set $$\mathcal {I}_l$$ describes the observations for which the prediction $$\hat{f}(\varvec{x}_i)$$ falls into bin $$b_l$$: $$\mathcal {I}_l = \{i \in \{1, \dots , n\}\ |\ \hat{f}(\varvec{x}_i)\in b_l\}$$. A probabilistic classifier $$\hat{f}$$ is well-calibrated if the points $$p_{\text {cal},l}$$ are close to the bisector.

In the distributed setup, the points $p_{\text {cal},l}$ are constructed by applying the distributed mean to both points for each bin at each site:

1. Set all $b_1, \dots , b_{n_{\text {bin}}}$, and communicate them to the sites.
2. Calculate the values $c_{l, \text {pf}}^{(k)} = a_{\text {sum}}(\{\hat{f}(\varvec{x}_i^{(k)})\ |\ i \in \mathcal {I}_l^{(k)}\})$ and $c_{l, \text {tf}}^{(k)} = a_{\text {sum}}(\{y_i^{(k)}\ |\ i \in \mathcal {I}_l^{(k)}\})$ for all $l = 1, \dots , n_{\text {bin}}$.
3. Send $\{(c_{l, \text {tf}}^{(k)}, c_{l, \text {pf}}^{(k)}, |\mathcal {I}^{(k)}_l|)\ |\ k = 1, \dots , K, l = 1, \dots , n_{\text {bin}}\}$ to the host if $|\mathcal {I}_l^{(k)}| \ge q$.
4. The host calculates the calibration curve $p_{\text {cal},l}$ by aggregating the elements $\text {tf}_l = (\sum \nolimits _{k=1}^K |\mathcal {I}_l^{(k)}|)^{-1}\sum \nolimits _{k=1}^K c_{l,\text {tf}}^{(k)}$ and $\text {pf}_l = (\sum \nolimits _{k=1}^K |\mathcal {I}_l^{(k)}|)^{-1}\sum \nolimits _{k=1}^K c_{l,\text {pf}}^{(k)}$ for $l = 1,\dots ,n_{\text {bin}}$.

#### The distributed ROC-GLM

**Fig. 2:**
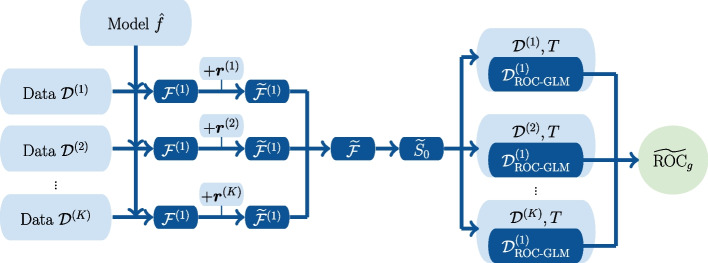
The distributed ROC-GLM procedure (distrROCGLM) calculates the distributed approximation $\widetilde{\text {ROC}}_g$ of $\text {ROC}_{g}$. The sites (here $K = 3$) communicate scores with added noise. Centrally, the global negative survivor function $\tilde{S}_{\bar{D}}$ is determined and returned to the sites. Finally, the distributed probit regression operates on local intermediate data $\mathcal {D}_{\text {ROC-GLM}}^{(k)}$

Two aspects are of relevance when building the distributed version of the ROC-GLM (distrROCGLM): (1) The distributed version of the empirical survivor function and (2) a distributed version of the probit regression. Figure 2 shows details of the general procedure. The starting point of the distributed ROC-GLM is the private data $\mathcal {D}^{(1)}, \dots , \mathcal {D}^{(K)}$ on the *K* sites.

The global survivor function $\hat{S}_{0}$ is approximated by $\tilde{S}_{0}$ (Approximating the global survivor functions section) using principle **A2**. The computation of $\tilde{S}_{0}$ depends on the level of privacy induced by the $(\varepsilon ,\delta )$ DP parameters (Differential privacy section). The accuracy of the AUC as well as its CI depends on the choice of $\varepsilon$ and $\delta$. The global survivor function $\tilde{S}_{0}$ is transmitted to each of the *K* sites and allows calculation of a local version of the intermediate data set $\mathcal {D}_{\text {ROC-GLM}}^{(k)}$ (see The ROC-GLM section). The distributed probit regression complies with principle **A1** and produces the distributed ROC-GLM parameter estimates (see Distributed GLM section). Using the ROC-GLM of these parameters, denoted by $\widetilde{\text {ROC}}_g$, allows calculation of the approximated AUC, denoted by $\widetilde{AUC}_{\text {ROC-GLM}} = \int _0^1 \widetilde{\text {ROC}}_g(t|\hat{\varvec{\gamma }})\ dt$. Finally, the CIs can be calculated based on a variance estimation, which also complies with principle **A2** (see Distributed CIs for the AUC based on the Score function section).

#### The distributed GLM model building

Distributed GLM section describes the federation of the Fisher Scoring algorithm and explains how it can be applied under principle **A2**. Therefore, distributed privacy protected GLM model building does not pose specific challenges.

### Approximating the global survivor functions

The privacy-preserving calculation of the global negative survivor function $\hat{S}_{0}$ needs special attention. It is prohibited to directly communicate score values $\mathcal {F}_{0}^{(k)}$ from the local sites to the central analyst. Instead, we propose to calculate an approximation $\tilde{S}_{0}$: First, we determine the $\ell _2$-sensitivity of the prediction model $\hat{f}$ and set the value of $\varepsilon$ and $\tau$. Then, we generate a noisy representation $\tilde{\mathcal {F}}_{0}^{(k)} = \mathcal {F}_{0}^{(k)} + \varvec{r}^{(k)}$ of the original score values $\mathcal {F}_{0}^{(k)}$ at each site. Second, the noisy scores are communicated to the host and pooled to $\tilde{\mathcal {F}}_{0} = \bigcup _{k=1}^K \tilde{\mathcal {F}}_{0}^{(k)}$ to calculate an approximation $\tilde{S}_{0}$ of the global survivor function. Third, $(\varepsilon , \delta )$ DP allows sharing $\tilde{S}_{0}$ with all sites. Forth, the local sites calculate the global placement values and create the intermediate data set used by the distributed probit regression.

### Distributed GLM

For distributed calculation of the GLM, we use an approach described by [14] and adjust the optimization algorithm of GLMs – the Fisher scoring – at its base to estimate parameters without performance loss. This approach complies with **A1**.

The basis of the ROC-GLM is a probit regression (and therefore a GLM) with $\mathbb {E}(Y\ |\ X = x) = g(x^{\textsf{T}} \theta )$ and link function *g*, response variable *Y*, and covariates *X*. The Fisher scoring is an iterative descending technique $\hat{\theta }_{m+1} = \hat{\theta }_m + \mathcal {I}^{-1}(\hat{\theta }_m)\mathcal {V}(\hat{\theta }_m)$ that uses second order gradient information. The components are the score vector $\mathcal {V}(\hat{\theta }_m) = [ \partial \ell _\theta (y,x) / \partial \theta ]_{\theta = \hat{\theta }_m} \in \mathbb {R}^{p}$ and the observed Fisher information $\mathcal {I}(\hat{\theta }_m) = [\partial \mathcal {V}(\theta ) / \partial \theta ]_{\theta = \hat{\theta }_m} \in \mathbb {R}^{p\times p}$ based on the log likelihood $\ell _\theta (\mathcal {D}) = \sum \nolimits _{i=1}^n \log (f_Y(y_i,x_i))$. A common stop criterion (as used in R function glm [22]) to determine whether the Fisher scoring has converged is when the relative improvement $|dev_m - dev_{m-1}| / (|dev_m| + 0.1)$ of the deviance $dev_m = -2\textsf{ln}( \ell _{\hat{\theta }_m}(\mathcal {D}))$ is smaller than a value *a*. The default value used in the glm function of R is $a = 10^{-8}$.

Sufficiently large non-overlapping data at the *K* sites (each subject contributes information only at a unique site) implies the additive structure of the global score vector $\mathcal {V}(\theta _m)$ and Fisher information $\mathcal {I}(\theta _m)$. With the site-specific score vector $\mathcal {V}_k(\theta _m)$ and Fisher information $\mathcal {I}_k(\theta _m)$, it holds:

6 $$\begin{aligned} \mathcal {V}(\hat{\theta }_m) & = \sum \limits _{k=1}^K \mathcal {V}_k(\hat{\theta }_m) \end{aligned}$$

7 $$\begin{aligned} \mathcal {I}(\hat{\theta }_m) & = \sum \limits _{k=1}^K \mathcal {I}_k(\hat{\theta }_m) \end{aligned}$$

### Distributed CIs for the AUC based on the Score function

The distributed calculation of the global sample mean ($\text {distrAVG}(\varvec{v}^{(1)}, \dots , \varvec{v}^{(K)})$) complies with **A1** as well as the distributed version of the sample variance $\widehat{\textsf{var}}(\varvec{v}) = (n-1)^{-1} \sum \nolimits _{i=1}^n (v_i - \bar{v})^2$. In the first step, the sample mean is calculated using $\bar{v} = \text {distrAVG}(\varvec{v}^{(1)}, \dots , \varvec{v}^{(K)})$ and shared with all *K* sites. In the second step, each site calculates the aggregation $a_{\text {var}}(\varvec{v}^{(k)}) = \sum \nolimits _{i=1}^{n^{(k)}} (v_i^{(k)} - \bar{v})^2$, which is further aggregated to the sample variance $\widehat{\textsf{var}}(\varvec{v}) = (n - 1)^{-1}\sum \nolimits _{k=1}^K a_{\text {var}}(\varvec{v}^{(k)})$: $\text {distrVAR}(\varvec{v}^{(1)}, \dots , \varvec{v}^{(K)})$. The operations $\text {distrAVG}$ and distrVAR fulfill **A1** if $n^{(k)} \ge q$, $\forall k \in \{1, \dots , K\}$.

The operation distrVAR provides a non-disclosing distributed CIs for the global AUC. As described in Empirical calculation of the ROC curve and AUC and CI for the empirical AUC sections, the calculation of the approximated CI requires both approximated survivor functions $\tilde{S}_{0}$ and $\tilde{S}_{1}$ (see Approximating the global survivor functions section). A distributed CI $\widetilde{\textsf{ci}}_\alpha$ to approximate $\textsf{ci}_{\alpha }$ follows from Formula (3).

## Simulation study

### General considerations

The aim of the simulation study is to understand the effect of the noise (introduced by DP) on the AUC estimate of the distributed ROC-GLM and its DeLong confidence intervals. We take the global empirical AUC [11, 17] as a proxy for the true AUC of the underlying data generating process. Our goal is not to construct better estimates for the true AUC, but to study the difference between our distributed approach to the empirical AUC on the the pooled data.

In this context, we assess the bias of the distributed approach and measure the difference $\Delta AUC = AUC - \widetilde{AUC}_{\text {ROC-GLM}}$ between the empirical *AUC* on pooled data (Empirical calculation of the ROC curve and AUC section) and the distributed ROC-GLM $\widetilde{AUC}_{\text {ROC-GLM}}$ (General principles section).

To evaluate CI related bias, we calculate the error $\Delta \textsf{ci}_{\alpha }$ based on the symmetric difference between $\textsf{ci}_{\alpha }$ proposed by DeLong et al. ([6], see Sect. 3.3) and our non-disclosing distributed approach $\widetilde{\textsf{ci}}_{\alpha }$ (Distributed CIs for the AUC based on the Scor function section). We study $\Delta \textsf{ci}_{\alpha } = |\widetilde{\textsf{ci}}_{\alpha , l} - \textsf{ci}_{\alpha , l}| + |\widetilde{\textsf{ci}}_{\alpha , r} - \textsf{ci}_{\alpha , r}|$, with indices *l* and *r* denoting the left and right side of the CI, respectively.

We explore the following research questions:

- **Question 1– Correctness of the AUC inferred from ROC-GLM and distributed ROC-GLM.**(Correctness of the AUC inferred from ROC-GLM and distributed ROC-GLM section): Which privacy parameters $\varepsilon$ and $\delta$ result in $|\Delta AUC|$ below 0.01?
- **Question 2–Correctness of the AUC CIs inferred from ROC-GLM and distributed ROC-GLM.** (Correctness of the AUC CIs inferred from ROC-GLM and distributed ROC-GLM section): Which privacy parameters $\varepsilon$ and $\delta$ result in $\Delta \textsf{ci}_{\alpha }$ below 0.01?

### Data generation

In order to avoid the specification of the score distributions in both outcome groups, we simulate data as follows. We generate uniformly distributed AUC values between 0.5 and 1. (1) The population size *n* is randomly chosen from $\{100, 200, \dots , 2500\}$. (2) For each $i \in \{1, \dots , n\}$, the *true* prediction scores are generated from the uniform distribution $\mathcal {F}_i\sim U[0,1]$. Next, (3) the class membership $y_i\in \{0,1\}$ is determined by $y_i = \mathbb {1}(\mathcal {F}_i \ge 0.5)$. This results in a perfect discrimination by scores between positives and negatives (AUC=1). (4) The perfect ordering of the class values with respect to individual scores is broken by flipping labels randomly. A set of indexes $\mathcal {I}$ of size $\lfloor \gamma n \rfloor$ is selected for which the corresponding labels are replaced by $y_i \sim \text {Ber}(0.5)$, $\forall i \in \mathcal {I}$. The fraction $\gamma$ is sampled from a *U*[0.5; 1] distribution. (5) For comparison, the empirical AUC is calculated from the vector of scores $\mathcal {F}$ and flipped labels *y*. (6) The non-disclosing distributed process described in General principles section is based on 5 centers and produces the $\widetilde{AUC}_{\text {ROC-GLM}}$ and $\widetilde{ci}_{0.05}$. The examined values for the distributed ROC-GLM are described in Correctness of the AUC inferred from ROC-GLM and distributed ROC-GLM section. The simulation is repeated $N^{sim} = 10000$ times.

**Fig. 3:**
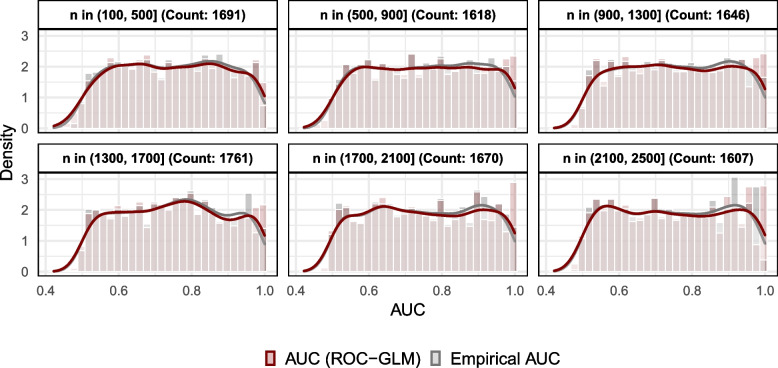
Densities of 10 000 simulated values of the empirical and non-distributed ROC-GLM AUC. The Densities are grouped according data sizes *n*

Figure 3 shows the empirical distribution of the empirical as well as ROC-GLM-based AUC values depending on the sizes of *n*. The distribution of the empirical AUC values is close to the uniform distribution over the range of 0.5 to 1. The behaviour of AUC estimates at the borders can be explained as follows: To obtain an AUC value of one, it is necessary to keep all original class labels *y*. However, this happens rarely, due to the randomized assignment of the observations chosen in $\mathcal {I}$. The same applies to AUC values close to 0.5. An AUC value of 0.5 appears if the class labels are completely randomized. This is also a rare event.

### Results

#### Correctness of the AUC inferred from ROC-GLM and distributed ROC-GLM

##### ROC-GLM

Figure 3 shows a nearly perfect overlap of the means of the simulated empirical as well as the non-distributed ROC-GLM AUC values in the range of values between 0.6 and 0.8. Nevertheless, the behaviour at the right border results from numerical problems of the probit regression on data containing only very few values of zero and mostly values of 1.

Table 1 shows summary statistics of $(AUC - AUC_{\text {ROC-GLM}})$ organized by bins of the empirical AUC of width 0.025. In **Question 1**, an absolute difference below 0.01 is requested, which is fulfilled over the whole AUC range. Mean and median differences ranging from 0.5 to 0.95 fulfill this requirement, whereas for empirical AUC values between 0.95 and 0.975 slightly larger differences are observed. Moreover, for the lower bins, the difference is always positive while it is negative for the higher bins. This is in line with the example from Appendix A.6 for biased ROC-GLM estimation.

**Table 1:** Minimum, 0.25-quantile/1st quantile, median, mean, 0.75-quantile/3rd quantile, maximum, standard deviation, and the differences $AUC - AUC_{\text {ROC-GLM}}$ of the bins containing the respective subset of the 10000 empirical AUC values

Emp. AUC (Bin)	Min.	1st Qu.	Median	Mean	3rd Qu.	Max.	Sd.	Count
(0.5, 0.525]	$-0.0044$	$-0.0001$	0.0005	0.0040	0.0014	0.0506	0.0100	431
(0.525, 0.55]	$-0.0052$	0.0001	0.0006	0.0027	0.0011	0.0986	0.0123	505
(0.55, 0.575]	$-0.0031$	0.0003	0.0009	0.0014	0.0015	0.1298	0.0080	465
(0.575, 0.6]	$-0.0018$	0.0006	0.0012	0.0015	0.0017	0.1567	0.0072	482
(0.6, 0.625]	$-0.0044$	0.0009	0.0015	0.0014	0.0020	0.0064	0.0010	485
(0.625, 0.65]	$-0.0039$	0.0012	0.0017	0.0017	0.0022	0.0069	0.0010	501
(0.65, 0.675]	$-0.0031$	0.0013	0.0018	0.0018	0.0023	0.0068	0.0011	503
(0.675, 0.7]	$-0.0022$	0.0012	0.0018	0.0018	0.0023	0.0064	0.0010	465
(0.7, 0.725]	$-0.0082$	0.0010	0.0016	0.0016	0.0023	0.0070	0.0012	523
(0.725, 0.75]	$-0.0031$	0.0008	0.0015	0.0014	0.0021	0.0087	0.0012	485
(0.75, 0.775]	$-0.0058$	0.0004	0.0011	0.0010	0.0018	0.0053	0.0013	501
(0.775, 0.8]	$-0.0053$	$-0.0003$	0.0004	0.0005	0.0012	0.0088	0.0015	523
(0.8, 0.825]	$-0.0061$	$-0.0013$	$-0.0002$	$-0.0004$	0.0005	0.0045	0.0016	476
(0.825, 0.85]	$-0.0125$	$-0.0023$	$-0.0013$	$-0.0014$	$-0.0003$	0.0059	0.0019	484
(0.85, 0.875]	$-0.0111$	$-0.0037$	$-0.0026$	$-0.0025$	$-0.0014$	0.0074	0.0020	520
(0.875, 0.9]	$-0.0136$	$-0.0056$	$-0.0044$	$-0.0043$	$-0.0030$	0.0076	0.0023	534
(0.9, 0.925]	$-0.0195$	$-0.0080$	$-0.0065$	$-0.0065$	$-0.0052$	0.0066	0.0026	515
(0.925, 0.95]	$-0.0193$	$-0.0105$	$-0.0091$	$-0.0089$	$-0.0076$	0.0056	0.0030	481
(0.95, 0.975]	$-0.0227$	$-0.0138$	$\varvec{-0.0113}$	$\varvec{-0.0113}$	$-0.0093$	0.0067	0.0037	503
(0.975, 1]	$-0.0180$	$-0.0093$	$-0.0062$	$-0.0064$	$-0.0034$	0.0013	0.0039	529

Bold values indicate that these AUC bins show absolute differences larger 0.01 and provide a negative answer to **Question 1**. The count column indicates the number of simulated AUC values per bin

**Fig. 4:**
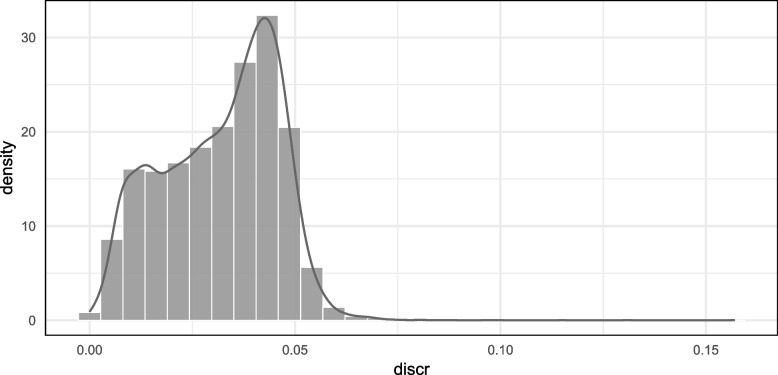
Distribution of area between the empirical ROC curve and the ROC-GLM curve. The distribution of the alternative discrepancy measure $discr_s$ is estimated from all simulated datasets $s \in \{1,\dots ,N^{sim}\}$

The results suggest that there are systematic deviations. Thus, we use as an alternative measure the $\ell _1$-norm that quantifies the discrepancy between the estimated empirical and the estimated GLM formulation of the ROC curve: the (absolute) area between both curves over the whole range *t*, that is, $discr_s = \int _0^1 |\text {ROC}_{g}(t | \hat{\varvec{\gamma }}) - \hat{S}_{1}(\hat{S}_{0}^{-1}(t))| dt$ for a data set $s \in \{1,\dots ,N^{sim}\}$. Figure 4 shows the empirical distribution of the defined measure over all simulations.

It can be seen that the difference are in general less than 5% (mean: 0.032, 25%- quantile: 0.021, 75% -quantile: 0.043). The small discrepancy with respect to the AUC is explained by the fact that there are areas where the empirical AUC is above the ROC-GLM and vice versa which compensate each other (see for example the left panel of Fig. 9 and Figure S4 in the appendix where this regions can be seen).

##### Distributed ROC-GLM

In the following, we investigate the accuracy of the AUC estimated by the distributed ROC-GLM. The respective DP parameters ($\varepsilon$ and $\delta$) must be determined in such a way that the answer to **Question 1** is positive. The data are distributed over five sites: The simulated prediction scores $\mathcal {F}$ and true classes *y* are randomly split into $K = 5$ parts $\mathcal {F}^{(1)}, \dots , \mathcal {F}^{(5)}$ and $y^{(1)}, \dots , y^{(5)}$. Our simulation setting uses $\varepsilon \in A_\varepsilon = \{0.1, 0.5, 1, 5, 10\}$ and $\delta \in A_\delta = \{10^{-5}, 10^{-4}, 10^{-3}, 10^{-2}, 10^{-1}\}$. Due to the Gaussian mechanism, we must also take the $\ell _2$-sensitivity into account as the added noise depends on it. Since we do not have an analytical description of the score function $\hat{f}$, we can not determine $\Delta _2(\hat{f})$ explicitly in this simulation. We assume $\Delta _2(\hat{f})\in A_{\Delta _2(\hat{f})} = \{0.01, 0.1, 0.2, 0.3, 0.4\}$. For the simulation, each setting of the grid $A_\varepsilon \times A_\delta \times A_{\Delta _2(\hat{f})}$ is evaluated by simulating 10000 data sets (cf. Data generation section) and hence obtaining 10000 $\widetilde{AUC}_{\text {ROC-GLM}}$ values that are compared to the respective empirical AUC.

**Fig. 5:**
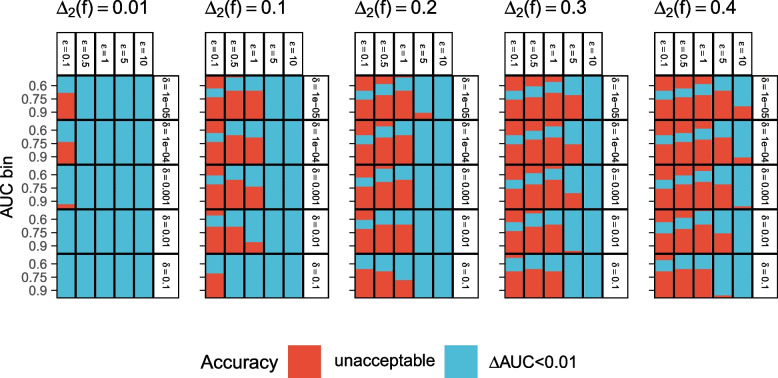
Absolute difference $|\Delta AUC|$ (mean absolute error, MAE): Combinations of privacy parameters ($\varepsilon$, $\delta$): Each rectangle contains empirical AUC bins of size 0.025 (cf. Table 1) and visualizes the mean of the absolute difference $|\Delta AUC|$ (mean absolute error, MAE) of the distributed ROC-GLM AUC compared to the empirical AUC per bin. Each rectangle corresponds to one simulation setting $(\Delta _2(\hat{f}), \varepsilon , \delta )$. The MAE per bin is categorized according to the required precision, with blue visualizing an $\text {MAE} \le 0.01$ (**Question 1**) while red shows an unacceptable accuracy measured as MAE larger than 0.01

Figure 5 shows the simulation results for different ($\varepsilon$, $\delta$) combinations. The absolute difference of $(AUC - AUC_{\text {distributed ROC-GLM}})$ is checked for having a value below 0.01. The results are based on 10000 simulation runs for 25 ($\varepsilon , \delta$) combinations and for each $\Delta _2(\hat{f})\in \{0.01, 0.1, 0.2, 0.3, 0.4\}$. The variance of the added noise to the scores is determined by the analytic Gaussian mechanism from [2]. The figure reveals that the bias between empirical and distributed ROC-GLM AUC depends heavily on the $\ell _2$-sensitivity. The smaller the sensitivity, less noise is required to ensure a certain level of privacy. Correspondingly, smaller choices of privacy parameters can and should be used to ensure privacy. Very small values of $\varepsilon$ lead often to unreliable results (except for a very small $\Delta _2(f)$ in combination with higher values of $\delta$). For larger values of $\varepsilon$ the results depend (besides the sensitivity) on $\delta$. For instance, the evaluation of the AUC on an algorithm with sensitivity $\Delta _2(f) = 0.1$ and $\varepsilon = 0.5$ would only be reliable with a very high value of $\delta = 0.1$ while a value of $\delta = 10^{-5}$ would be possible for $\Delta _2(f) = 0.01$ with $\varepsilon = 0.5$. For higher values of $\Delta _2(f)$, one has to fall back to higher values of $\varepsilon$. For example, consider a hypothetical dataset with 5000 records and an algorithm with $\Delta _2(f) = 0.3$. In this case one has to accept $\varepsilon = 10$ to guarantee a reliable estimate of the AUC while $\delta$ should be set to a small value.

#### Correctness of the AUC CIs inferred from ROC-GLM and distributed ROC-GLM

**Fig. 6:**
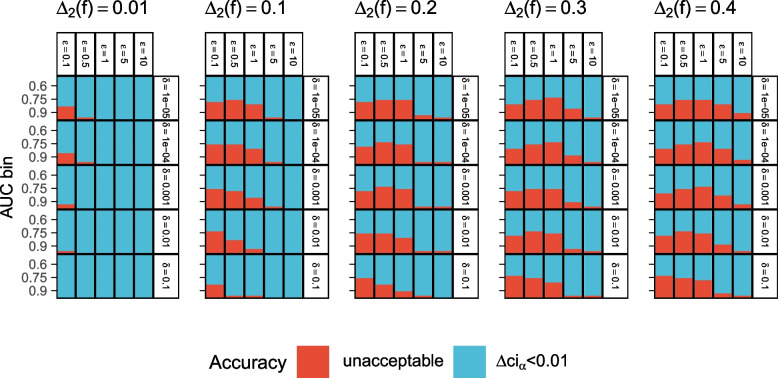
Mean relative error $\Delta \textsf{ci}_{0.05}$: Combinations of the privacy parameters $\varepsilon$ and $\delta$ and their applicability depending on $\Delta _2(\hat{f})$. Each rectangle contains empirical AUC bins of size 0.025 (cf. Table 1) and visualizes the mean of the relative error $\Delta \textsf{ci}_{0.05}$ of the distributed CI $\widetilde{ci}_{0.05}$ compared to $\textsf{ci}_{0.05}$. Blue shows accuracy values with $\Delta \textsf{ci}_{0.05} \le 0.01$ (**Question 2** applies), while red visualizes inaccuracies of $\Delta \textsf{ci} > 0.01$

The respective results in terms of acceptable $(\varepsilon , \delta )$ combinations are shown in Fig. 6. In general, acceptable $(\varepsilon , \delta )$ combinations under **Question 1** are also acceptable under **Question 2**. Therefore, we recommend using the more restrictive settings described in the previous Correctness of the AUC inferred from ROC-GLM and distributed ROC-GLM section for the AUC estimation of the distributed ROC-GLM.

## Data analysis

We develop a prognostic model on a pooled data and validate its predictive performance on a distributed test data set. We also compare the distributed validation results to results derived from the pooled analysis (see Comparison with pooled data section). As a privacy level, we choose a value of $q = 5$ (see General principles section, **A1**).

### About the data

The public data set from the German Breast Cancer Study Group [24] can be found in the TH.data package [12]. The dataset consists of records from 686 breast cancer patients to assess the effect of hormonal therapy on survival. Besides the binary variable hormonal treatment (horTH), the data contains information on age (age), menopausal status (menostat), tumor size (in mm, tsize), tumor grade (tgrade), number of positive nodes (pnodes), progesterone receptor (in fmol, progrec), estrogen receptor (in fmol, estrec), recurrence-free survival time (in days, time), and censoring indicator (0- censored, 1- event, cens).

We split the data into a training data ( 60 % , 412 observations) and split the remaining (40 %, 250 observations) into 5 parts $\mathcal {D}^{(1)}, \dots , \mathcal {D}^{(5)}$ with $n^{(1)} = 51$, $n^{(2)} = 45$, $n^{(3)} = 55$, $n^{(4)} = 46$, and $n^{(5)} = 53$ that are used for the distributed validation. The *f* of interest is $p(t|\varvec{x}) = P(T > t| X = \varvec{x})$: Probability of surviving time point *t* without recurrence based on covariates $\varvec{x}$. We choose $t = 730$ (two years). Since we evaluate the binary predictor *patient survives at least t days without recurrence*, we omit 24 patients censored before 730 days from the validation sets. As censoring is assumed to be independent and does not introduce selection bias. For both sets, train and test, roughly 25% of the observations encountered an event before 730 days. We provide the Kaplan-Meier curves of the used training and test data in Appendix A.4. The predicted scores are the survival probabilities $\hat{y_i} = {\hat{f}}(\varvec{x}_i) = \hat{p}(730|\varvec{x}_i)$ with $\varvec{x}_i\in \cup _{k=1}^K \mathcal {D}^{(k)}$. The corresponding binary variable $y_i$ equals 0 if the patient dies in [0, 730] or a recurrence was observed, and $y_i$ equals 1 if otherwise. Therefore, a high value for the survival probability $\hat{y}_i$ ideally corresponds to a binary outcome of 1.

### About the model

We choose a random survival forest [4, 13] using the R package ranger [29] as a prognostic model ${\hat{f}}$ for the survival probability $p(t|\varvec{x})$. With the exception of the number of trees (which is set to 20), the random forest was trained with the default hyperparameter settings of the ranger implementation. The model formula is given by

$$\begin{aligned} \textsf {Surv(time, cens)} \sim \textsf {horTh + age + tsize + tgrade + pnodes + progrec + estrec}. \end{aligned}$$

### About the implementation

The implementation is based on the DataSHIELD [10] framework and is provided by an R package called dsBinVal (github.com/difuture-lmu/dsBinVal). Further details about these methods and privacy considerations can be found in the respective GitHub README.

### Aim of the analysis

The main goal of the analysis is to test the hypothesis that the true AUC is significantly larger than 0.6 as the minimal prognostic performance of the model ${\hat{f}}$. The significance level is set to $\alpha = 0.05$:

8 $$\begin{aligned} H_0: \ AUC \le 0.6 \ \ \text {vs.} \ \ H_1: \ AUC > 0.6 \end{aligned}$$

To test the hypothesis, we estimate the AUC with $\widetilde{AUC}_{\text {ROC-GLM}}$ using the distributed ROC-GLM as well as the approximated CI $\widetilde{\textsf{ci}}_{0.05}$. We reject $H_0$ if $AUC > 0.6,\ \forall AUC \in \widetilde{\textsf{ci}}_{0.05}$.

### Analysis plan

In the following, (1) we start with the calculation of the $\ell _2$-sensitivity (Choice of the privacy parameters section). Depending on the result and the size of the data, we set the privacy parameters $\varepsilon$ and $\delta$ using the algorithm from [2]. Next, (2) we continue with fitting the distributed ROC-GLM and calculating the approximation of the AUC’s confidence interval (Calculation of the distributed ROC-GLM section). At this point, we are able to make a decision about the hypothesis in Eq. (8). In a final step, (3) we demonstrate how to check the calibration of the model using the distributed Brier score and calibration curve (Checking the calibration section).

### Choice of the privacy parameters

Given the model and the data set, the $\ell _2$-sensitivity is $\Delta _2(\hat{f}) = 0.178$. The results of Correctness of the AUC inferred from ROC-GLM and distributed ROC-GLM section, imply $\varepsilon = 5$ and $\delta = 0.01$ to obtain a reliable estimation.

### Calculation of the distributed ROC-GLM

**Fig. 7:**
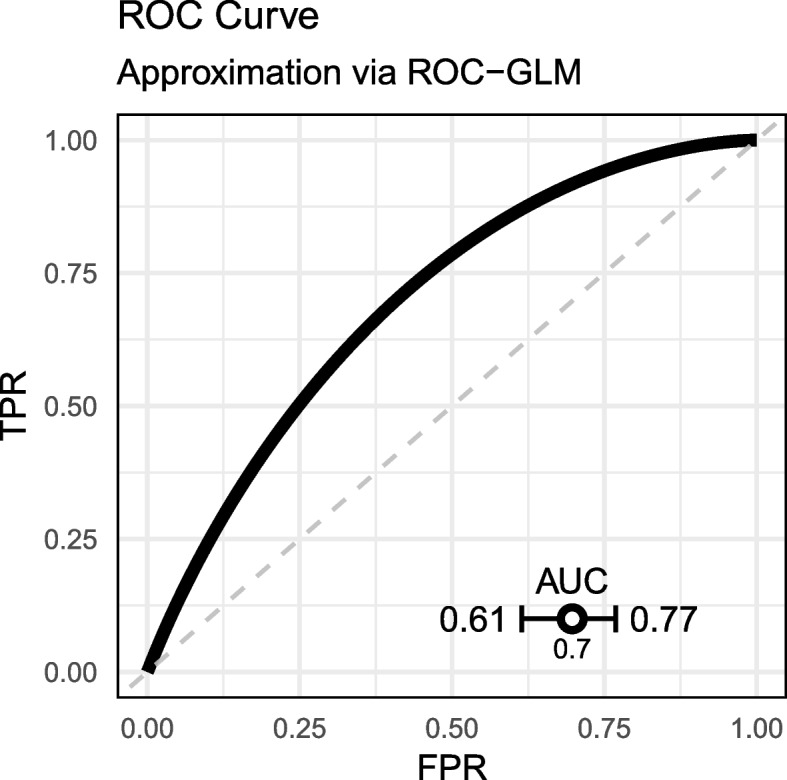
ROC curve estimated by the distributed ROC-GLM

The fit of the ROC-GLM results in parameter estimates of $\gamma _1 = 0.79$ and $\gamma _2 = 1.16$. The AUC obtained from the ROC curve using these parameters is $AUC_{\text {ROC-GLM}} = 0.697$ with $\widetilde{\textsf{ci}}_{0.05} = [0.615, 0.769]$. The results are visualized in Fig. 7.

Based on the given CI, we significantly reject $H_0$ for $H_1$ and hence assume the true AUC to be greater than 0.6.

### Checking the calibration

**Fig. 8:**
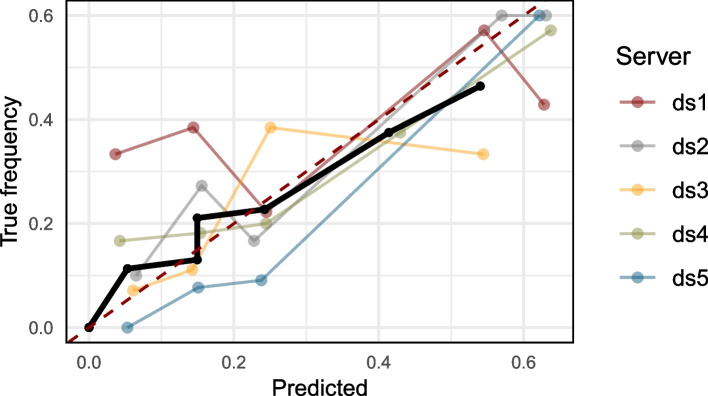
Distributed calibration curve (bold line) and calibration curves of the individual sites using 10 bins. Note that aggregated values from the site are only shared if one bin contains more than 5 values. See Appendix A.5 for tables containing the numbers of values per bin

The Brier score of ${\hat{f}}$ calculates to $\text {BS} = 0.184$ and indicates a good but not perfect calibration. We further assume our model to be not calibrated perfectly. Figure 8 shows the distributed calibration curve as well as the individual calibration curves per site. Furthermore, we observe that the range of the calibration curve does not cover the whole range of the scores ${\hat{f}}(x) \in [0, 1]$. This indicates that our model does not predict scores close to 1. We want to highlight that, due to privacy reasons, not all score values were included in the calculation; aggregated values are only shared if they consist of at least 5 elements. The table in Appendix A.5 shows the number of elements per bin and site.

### Comparison with pooled data

**Fig. 9:**
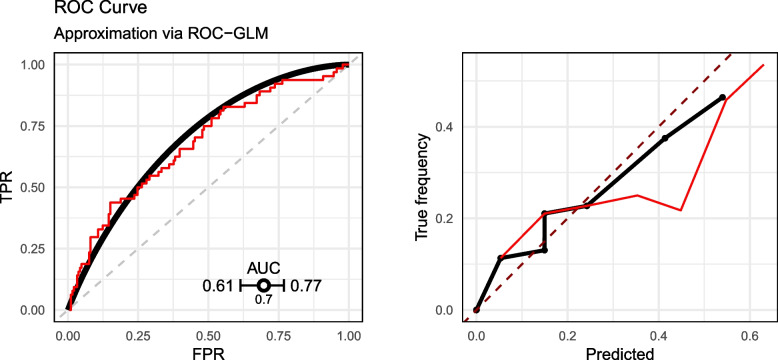
Comparison of the empirical ROC curve with ROC curve obtained by the distributed ROC-GLM (left). Comparison of the calibration curve when calculated on the pooled scores compared with the distributed calibration curve (right). The thin (red) curves are the lines on the pooled data

Comparison of both ROC curves (empirical ROC on the pooled sample and the distributed ROC-GLM) (Fig. 9, left) shows an acceptable fit of the ROC-GLM. However, by scrutinizing the plot more closely, one can see that there is a discrepancy between the empirical ROC curve and the estimated ROC-GLM: For a small FPR, the curve from the ROC-GLM is below the empirical one. On the other hand, a similar trend is observed for high values of the FPR in the opposite direction. This refers to differences also observed in the example in Appendix A.6. The resulting AUC values are $\widetilde{AUC}_{\text {ROC-GLM}} = 0.697$ and $AUC = 0.679$ with $|\Delta AUC| = 0.018$. The CIs of the approximated CI $\widetilde{\textsf{ci}}_{0.05} = [0.615, 0.769]$ and the CI on the pooled scores $\textsf{ci}_{0.05} = [0.598, 0.751]$ reveals a slightly more optimistic CI estimation in the distributed setup. The error of the CI calculates to $\Delta \textsf{ci}_{0.05} = 0.034$.

The distributed calibration curve shows an overlap with the calibration curve in areas where all data are allowed to be shared. For bins where this is not the case, the distributed calibration curve differ. Still, the tendency of over- or underestimation of the distributed calibration curve corresponds to one of the pooled curves. The bins for which the full information was received are [0, 0.1], (0.1, 0.2], and (0.2, 0.3] (cf. Appendix A.5 Table S1). For all other bins, at least one site was not allowed to share the aggregated values. The pooled calibration curve shows potential overprediction which is not is reflected by the distributed curve.

The Brier score of the pooled and distributed approach is equal.

## Reproducibility considerations

All experiments were conducted using R version 4.1.2 on a Linux machine with an Intel(R) Core(TM) i7-8665U CPU @ 1.90GHz processor. The package used to run the simulation was batchtools [15]. The code to reproduce all results as well as all simulation results is available in a GitHub repository^4^. The repository contains a README file with further details and a script to install all packages with the respective version used when the benchmark was conducted.

The code to conduct the data analysis is given in a separate GitHub repository^5^. The repository contains the data, an installation of all necessary packages, as well as code to set up the publicly available DataSHIELD server^6^ to run the analysis^7^.

## Discussion

Distributed non-disclosing (i.e., privacy-preserving) strategies for data analysis are highly relevant for data-driven biomedical research. Since the analyses can be considered anonymous, current legal data protection frameworks allow their use without requesting specific consent. Protecting privacy by appropriate means is fundamental when using personal data for research. Distributed approaches also enable taking part in broader network structures without additional administrative work concerning data protection issues. Privacy-preserving distributed computation allows researchers to digitally cooperate and leverage the value of their data while respecting data sovereignty and without compromising privacy. Besides the privacy preservation in algorithms that are backed up with security mechanisms, it is worth noting that software is also a key player in privacy-preserving analysis. For example, most models fitted with the statistical software R attach data directly to the model object. Sharing these objects without caution gives analysts direct access to the training data (cf., e.g., [23]).

International activity has been dedicated to setting up distributed non-disclosing analysis frameworks, which implement machine learning approaches into a distributed analysis scheme. However, our impression is that algorithms for distributed *validation* of these learning algorithms are lacking.

In this paper, we specifically focused on the assessment of discrimination and calibration of learning algorithms with a binary outcome. The discrimination is estimated by a ROC curve and its AUC. We also provide CIs to the distributed AUC estimate. The distributed estimation process is based on *placement values* and *survivor functions*. They represent qualities of the global distribution of score values (aggregated over all centers). To do this in a non-disclosing way, we applied differential privacy techniques. With the creation of the placement values and the transmission of this information to the local server, we applied a distributed version of the ROC-GLM approach to estimate the ROC curve and its AUC in a distributed way. We used a straightforward approach for the distributed GLM estimation. However, we acknowledge that there may be more efficient approaches.

The proposed method implements a combination of aggregation and differential privacy (DP) with privacy parameters ($\varepsilon , \delta$). DP offers a solution to exchange critical information privately to other sites, but a part of the information is lost through the induced noise of the privacy mechanism. The balance between utility (i.e. accurate estimates) and privacy must be carefully weighted. The results suggest, that for algorithms with a small sensitivity, the estimates stay reliable. However, for a higher sensitivity this is not the case. In general, a higher value of $\delta$ may lead to more flexibility (and therefore to a higher privacy level) with respect to $\varepsilon$, e.g. setting $\delta =0.1$. This suggests, that $\varepsilon$-DP is broken in 10% of the cases. It is questionable, whether this is an acceptable value.

We discuss broadly the potential bias in the approximation of the ROC curve by the distributed GLM approach and show results in Table 1 and Fig. 9. We focus on a binary measure of bias ($|\Delta AUC| < 0.01$) and did not address bias issues in detail. We did not explore how bias may be assessed by choosing different metrics (like relative measures). We did not explore aspects of unbalanced datasets and there effect on metrics like negative/positive predictive value. Hence, a more comprehensive analysis of the proposed method is necessary: even though the presented simulation studies provides valuable insights into the proposed method, it lacks of a in-depth detailed analysis. It is missing a comparison of the empirical ROC curve and its distributed ROC-GLM counterpart in terms of a $\ell _1$-metric.

Besides the potential bias of the ROC-GLM, the simulation study of the DP parameters considers only a selected range of configurations and does not further investigate their impact beyond the binary threshold. Additionally, the application limits itself only to one exemplary scenario with one dataset and one defined algorithm. Therefore, it can rather be seen as a didactic example. An in-depth examination of various classification tasks with different characteristics of data and classifiers under real-world conditions are necessary. Hence, future work is required to address the mentioned points in a comprising simulation study and a range of application settings.

Furthermore, a reviewer pointed to the potential anti-conservative effect of the proposed procedure. Figure 9 (left panel) suggests to reject the Null-hypothesis that the AUC is below 0.6 on a 5%-level while the result given in Comparison with pooled data section for the 95%-CI on the pooled data contains 0.6.

In view of these critical points, we therefore recommend applying the proposed method with caution at the moment. It is a straight-forward and pragmatic way to validate data in a federated manner while preserving privacy. Moreover we provide R code that directly implements the proposed method in the DataSHIELD framework. However, the previously mentioned problems imply, that the software should not be used as a black-box tool. It can serve as a low-level entry to investigate these issues for a specific setting.

We also want to highlight, that the proposed strategy cannot be used to develop a full machine learning model on distributed data. We focus exclusively on validating an already trained model, using data from other sites only once for this specific context. In general, applying a DP algorithm many times on the same data implies a higher privacy loss. See for example Section 3.5 in [9] about composition theorems in DP.

The procedure proposed can be summarized as follows: (1) The validation of an algorithm requires that it is known and can be shared. (2) The calculation of $\Delta _2(f)$ provides essential input to determine the DP setting. It can be derived from the data at hand and the algorithm under validation. The selection of the DP parameters $(\varepsilon , \delta )$ depends on the setting and use-case specific features. (3) The user has also to specify the level of privacy for the aggregation (i.e. the minimal number of unique values *q* to be shared aggregated) under project specific requirements. It is recommended to apply the proposed procedure on settings with large datasets at the different sites.

We mainly concentrate on the validation of a prediction model while the property of the ROC-GLM is not fully explored. We do not address specific features of the ROC-GLM estimates and ignore aspects of unbiasdness and consistency. We demonstrate that the approximation of the AUC by the distributed ROC-GLM estimates introduces bias which needs to be controlled and assessed. The approach creates a bias and needs a pragmatic assessment of whether it is acceptable or not. If the proposed approach is used in an analysis, this aspect must be clearly described in the corresponding analysis plan and its impact on the analysis must be discussed. Our example shows that the proposed approach produces an overly liberal result.

But, it can be seen as an advantage of the proposed strategy that the privacy protecting aspects are also helpful for subgroup analyses. Moreover, the proposed approach makes it straightforward to develop distributed privacy protected GLM based classification models since the log-likelihoods consist of site specific independent additive parts. The procedure described in Distributed GLM section can also be applied to federated privacy protecting model building activities in the family of generalized linear models.

### Supplementary Information

Supplementary Material 1.

## Data Availability

The simulated datasets generated during the current study are available on GitHub, https://github.com/difuture-lmu/simulations-distr-auc.

## Abbreviations

AUC: Area under the curve
CI: Confidence interval
DP: Differential privacy
FPR: False positive rate
GLM: Generalized linear model
IPD: Individual patient data
MII: Medical Informatics Initiative
ROC: Receiver operating characteristics
TPR: True positive rate

## Footnotes

1 www.medizininformatik-initiative.de

2 Note, that the estimation of a ROC-GLM is not unbiased in the non-normal case. We provide an illustrative counterexample in Appendix A.6. which uses gamma-distributed score values in the outcome groups.

3 In theory, multiple definitions of adjacent inputs exist. Throughout this article, adjacent inputs are based on a histogram representation $\tilde{\varvec{x}}\in \mathbb {N}^p$ and $\tilde{\varvec{x}}^\prime \in \mathbb {N}^p$ of two input vectors $\varvec{x}$ and $\varvec{x}^\prime$. Two inputs are adjacent if the $\ell _1$ norm of $\tilde{\varvec{x}}$ and $\tilde{\varvec{x}}^\prime$ is equal to one: $\text {adjacent}\ \varvec{x},\ \varvec{x}^\prime \ \Leftrightarrow \ \Vert \tilde{\varvec{x}} - \tilde{\varvec{x}}^\prime \Vert _1 = 1$ (cf., [9]).

4 github.com/difuture-lmu/simulations-distr-auc

5 github.com/difuture-lmu/datashield-roc-glm-demo

6 Available at opal-demo.obiba.org. The reference, username, and password are available at the OPAL documentation opaldoc.obiba.org/en/latest/resources.html in the “Types” section.

7 We cannot guarantee the functionality of the DataSHIELD server or if it will be publicly available forever. However, we keep the repository up-to-date by using continuous integration, which is triggered automatically every week. This system also reports errors that occur if the analysis cannot be conducted on the test server anymore. Further information can be found in the README file of the repository.

## Data availability

The example data and simulated datasets generated during the current study are available on GitHub.
