# Supplementary material for: Distributed non-disclosive validation of predictive models by a modified ROC-GLM
Source: BMC Med Res Methodol. 2024 Aug 29;24:190. doi: 10.1186/s12874-024-02312-4 (PMC11363434; doi:10.1186/s12874-024-02312-4)
Supplement: Supplementary file 1 — Supplementary Material 1. [file 12874_2024_2312_MOESM1_ESM.zip › appendix/figures/gaussian-mechanism0.05.pdf]

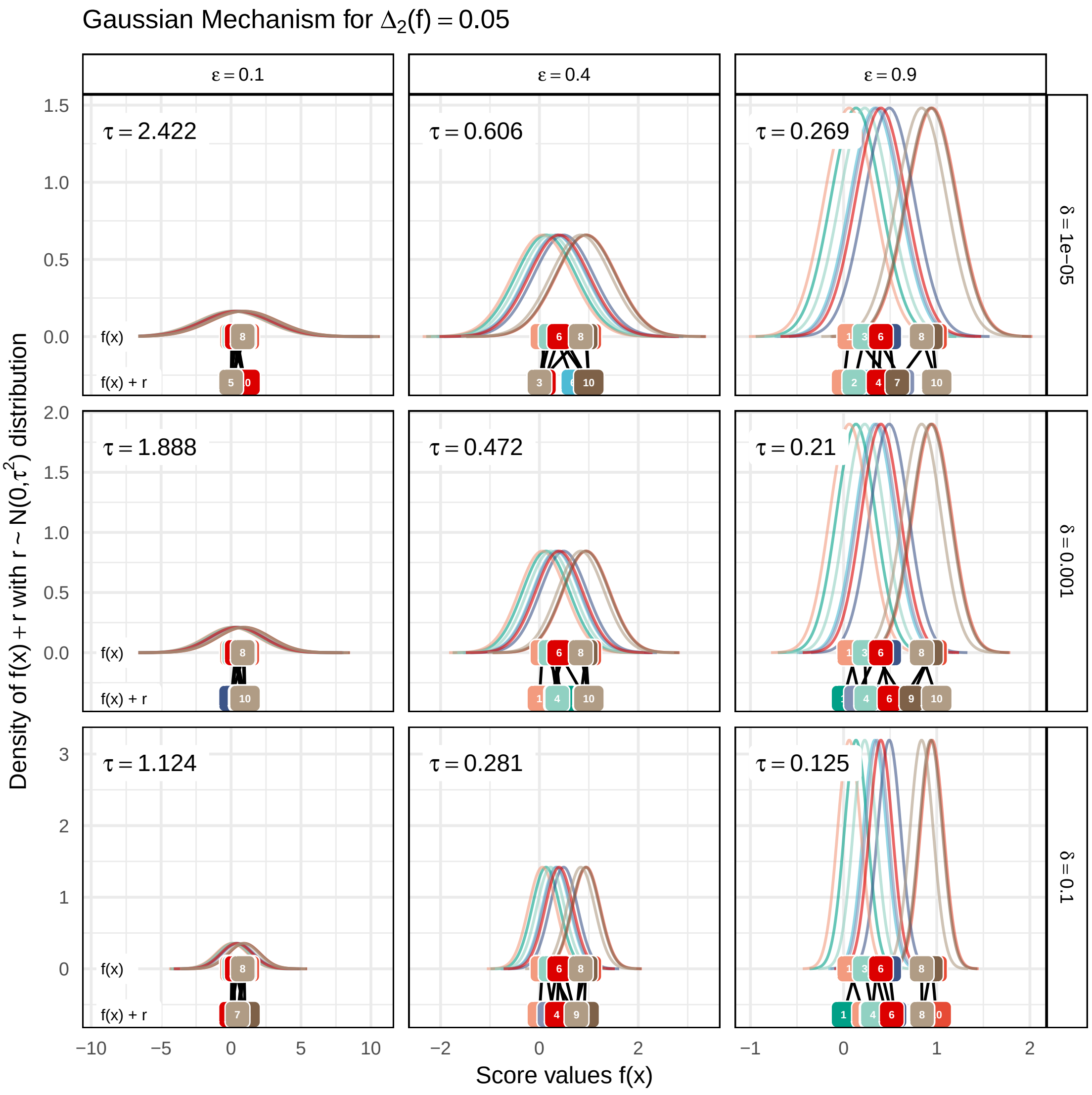

Gaussian Mechanism for Δ2(f) = 0.05
ε = 0.1
ε = 0.4
ε = 0.9
δ = 1e−05
δ = 0.001
δ = 0.1
τ = 2.422
τ = 0.606
τ = 0.269
τ = 1.888
τ = 0.472
τ = 0.21
τ = 1.124
τ = 0.281
τ = 0.125
f(x)
f(x) + r
Density of f(x) + r with r ~ N(0,τ2) distribution
Score values f(x)
