# Supplementary material for: Distributed non-disclosive validation of predictive models by a modified ROC-GLM
Source: BMC Med Res Methodol. 2024 Aug 29;24:190. doi: 10.1186/s12874-024-02312-4 (PMC11363434; doi:10.1186/s12874-024-02312-4)
Supplement: Supplementary file 1 — Supplementary Material 1. [file 12874_2024_2312_MOESM1_ESM.zip › appendix/appendix_revision2.pdf]

Supplementary Material for Distributed non-disclosive validation of predictive models by a modified ROC-GLM

Daniel Schalk[1,3,4], Raphael Rehms[2] Verena S. Hoffmann[2,3], Bernd Bischl[1,4] and Ulrich Mansmann[1,2,3]

[1] Department of Statistics, LMU Munich, Munich
[2] Institute for Medical Information Processing, Biometry and Epidemiology, LMU Munich, Munich
[3] DIFUTURE (DataIntegration for Future Medicine, www.difuture.de), LMU Munich, Munich
[4] Munich Center for Machine Learning (MCML), LMU Munich, Munich

Appendix

A.1. Decomposition of score vector and Fisher information

$$
\begin{aligned}
\mathcal{V}(\hat{\theta}_m) &= \left[\frac{\partial \ell_\theta(\mathcal{D})}{\partial \theta}\right]_{\theta=\hat{\theta}_m} = \left[\frac{\partial \sum_{i=1}^n \log(f_Y(y_i, x_i))}{\partial \theta}\right]_{\theta=\hat{\theta}_m} \\
&= \left[\sum_{i=1}^n \frac{\partial \log(f_Y(y_i, x_i))}{\partial \theta}\right]_{\theta=\hat{\theta}_m} = \sum_{k=1}^K \left[\sum_{(y,x)\in\mathcal{D}^{(k)}} \frac{\partial \log(f_Y(y, x))}{\partial \theta}\right]_{\theta=\hat{\theta}_m} \\
&= \sum_{k=1}^K \left[\frac{\partial \log(\ell_\theta(\mathcal{D}^{(k)}))}{\partial \theta}\right]_{\theta=\hat{\theta}_m} = \sum_{k=1}^K \mathcal{V}_k(\hat{\theta}_m)
\end{aligned}
$$

$$
\begin{aligned}
\mathcal{I}(\hat{\theta}_m) &= \left[\frac{\partial \mathcal{V}(\theta)}{\partial \theta}\right]_{\theta=\hat{\theta}_m} = \left[\frac{\partial \sum_{k=1}^K \mathcal{V}_k(\hat{\theta}_m)}{\partial \theta}\right]_{\theta=\hat{\theta}_m} = \\
&= \sum_{k=1}^K \left[\frac{\partial \mathcal{V}_k(\hat{\theta}_m)}{\partial \theta}\right]_{\theta=\hat{\theta}_m} = \sum_{k=1}^K \mathcal{I}_k(\hat{\theta}_m)
\end{aligned}
$$

A.2. Illustration of the Gaussian mechanism

The Gaussian mechanism adds zero-mean noise to a deterministic algorithm, i.e. $\mathcal{M}(\boldsymbol{x}) = \hat{f}(\boldsymbol{x}) + r$ (**?**). The noise r added in the Gaussian mechanism to obtain a noise representation of scores $\hat{f}(x)$ highly depends on the three values of $\Delta_2(f)$, ε, and δ. In our approach, we first examine the value of $\Delta_2(f)$ and then set ε and δ accordingly to not introduce too much noise and hence worsen the accuracy. Figure **??** and Figure **??** visualize the Gaussian mechanism for $\Delta_2(f) \in \{0.01, 0.05\} = 0.01$, $\varepsilon \in \{0.1, 0.4, 0.9\}$ and $\delta \in \{10^{-5}, 10^{-3}, 10^{-1}\}$ to get a rough idea on how the noise distorts the score values $f(x)$. The figures contain 10 exemplary score values (upper labels from 1 to 10), the value of the variance τ^2 (which is based on $\Delta_2(f)$, ε, and δ), the respective density of the normal distribution, and the noisy score values (lower labels). Both figures also visualizes how the Gaussian mechanism changes the order of the scores depending on the noise.

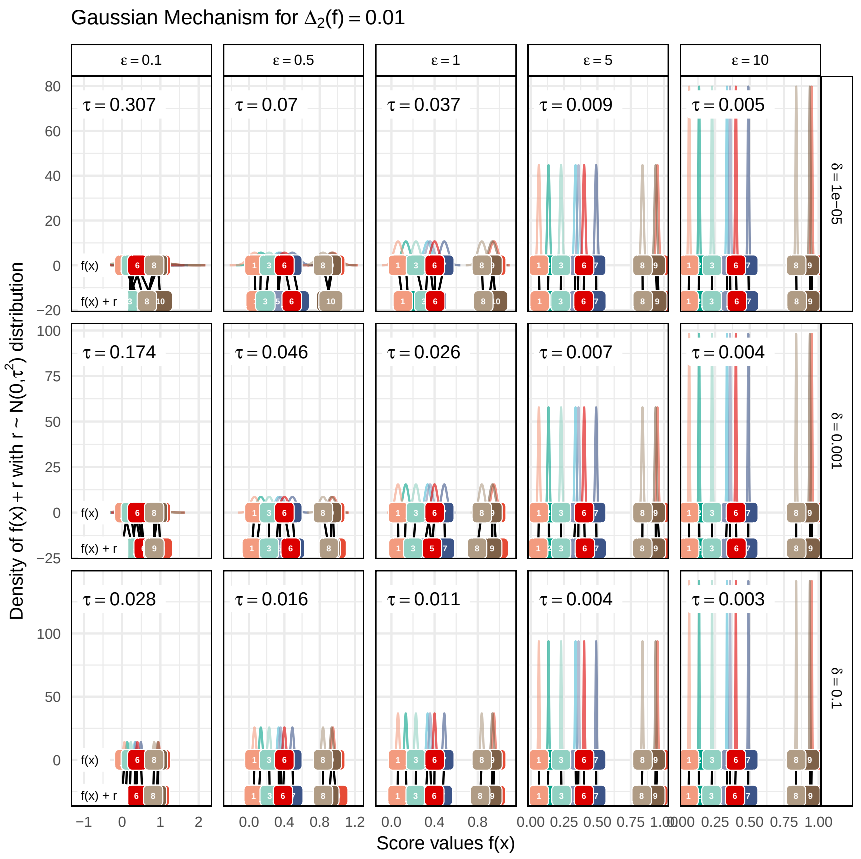

Figure S1: Visualization of how the Gaussian mechanism adds noise to the original score values $f(x)$ (upper labels) to obtain a noisy representation $f(x) + r$ (lower labels) with $r \sim \mathcal{N}(0, \tau^2)$. At each point in $f(x)$, the corresponding density of $N(f(x), \tau^2$ is added to visualize how the Gaussian mechanism shuffles the order (lines between the two labels of $f(x)$ and $f(x) + r$) of the score values, depending on the variance τ^2. The whole mechanism depends on the ℓ_2-sensitivity, which is set to 0.01 here.

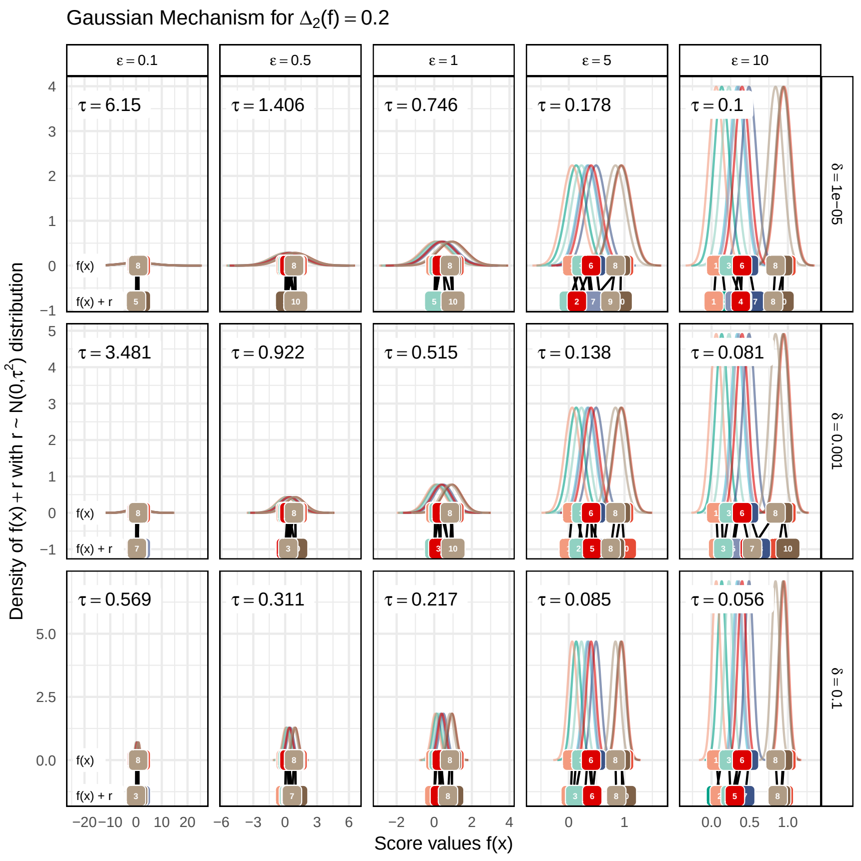

Figure S2: Visualization of how the Gaussian mechanism adds noise to the original score values $f(x)$ (upper labels) to obtain a noisy representation $f(x) + r$ (lower labels) with $r \sim \mathcal{N}(0, \tau^2)$. At each point in $f(x)$, the corresponding density of $N(f(x), \tau^2$ is added to visualize how the Gaussian mechanism shuffles the order (lines between the two labels of $f(x)$ and $f(x) + r$) of the score values, depending on the variance τ^2. The whole mechanism depends on the ℓ_2-sensitivity, which is set to 0.05 here.

A.3. Intuition of differential privacy

We present intuition of differential privacy as described in (**?**). Suppose an attacker has a prior p over two adjacent $\boldsymbol{x}, \boldsymbol{x}' \in \mathcal{X}$. A mechanism $\mathcal{M}$ now outputs a value $\xi \in \mathcal{Y}$. For ε-DP, the posterior odds satisfy the following statement:

$$\frac{p(\boldsymbol{x}|\xi)}{p(\boldsymbol{x}'|\xi)} \leq \exp(\varepsilon)\frac{p(\boldsymbol{x})}{p(\boldsymbol{x}')}$$

It can be interpreted as follows: The odds observing $\boldsymbol{x}$ or $\boldsymbol{x}'$ would not change more than $\exp(\varepsilon)$ after observing output ξ, or speaking for informally, the attacker has a maximal knowledge gain of $\exp{(\varepsilon)}$. We also want to hint on **?** to get an idea of used values in practical applications. The idea of the list stems from **?** to get an unified database of actual chosen parameters depending on the context. The described interpretation of ε implies that higher values could be critical. In practical settings high values are chosen very often. For example **?** propose a framework which implies that high values could be defended for various cases.

A.4. Kaplan-Meier curves of the `TH.data`

The used data set used in section 6 from the German Breast Cancer Study Group (?) can be found in the `TH.data` package (?). Figure ?? depicts the used data for the described task. We omit 24 data points in the test data as they encountered a censoring event before 2 years. Not deleting the entries would bias the result.

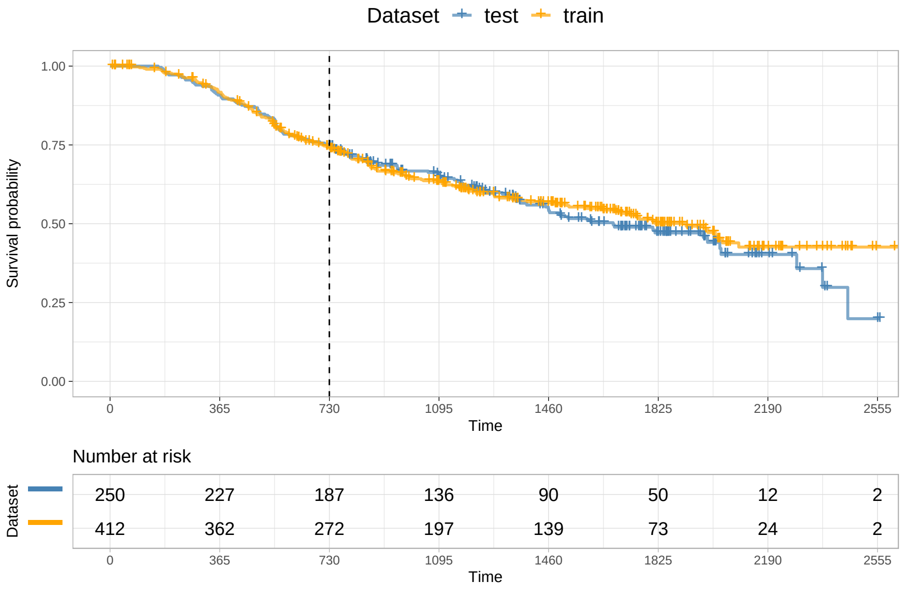

Figure S3: Kaplan-Meier curves for the training and the test data. The vertical line defines the 2-years which is used to cast the data into a binary classification task.

A.5. Number of observations per bin for the calibration curves

Site k	Bin									
	(0,.1]	(.1,.2]	(.2,.3]	(.3,.4]	(.4,.5]	(.5,.6]	(.6,.7]	(.7,.8]	(.8,.9]	(.9,1]
1	**6**	**13**	**9**	4	3	**7**	**7**	1	0	0
2	**10**	**11**	**6**	2	4	**5**	**5**	0	0	0
3	**14**	**9**	**13**	3	4	**6**	4	1	0	0
4	**12**	**11**	**5**	1	**8**	2	**7**	0	0	0
5	**11**	**13**	**11**	2	4	4	**5**	1	0	0
$\sum$	**53**	**57**	**44**	**12**	**23**	**24**	**28**	3	0	0

Table S1: Number of observations per bin. Values in these bins are shared only if the numbers per bin are larger than 5. The values for which this applies are highlighted as bold numbers.

A.6. Counterexample ROC-GLM

The ROC-GLM may be biased for non-normal distributed scores when the shape of the positives and negatives are different. **?** argues that the ROC-GLM can serve as a good estimator of the ROC curve, even if the distribution of the scores is non-normal. Here, we give a short counterexample that this can cause problems. We simulate data according to two skewed Gamma distributions and compare the results for the ROC-GLM (estimated in a non-federated setting) form with the empirical ROC curve. The phenomena described in the main paper can also be seen in Figure **??**. We also provide the code within this document to have a minimal version and get a basic idea of the general problem.

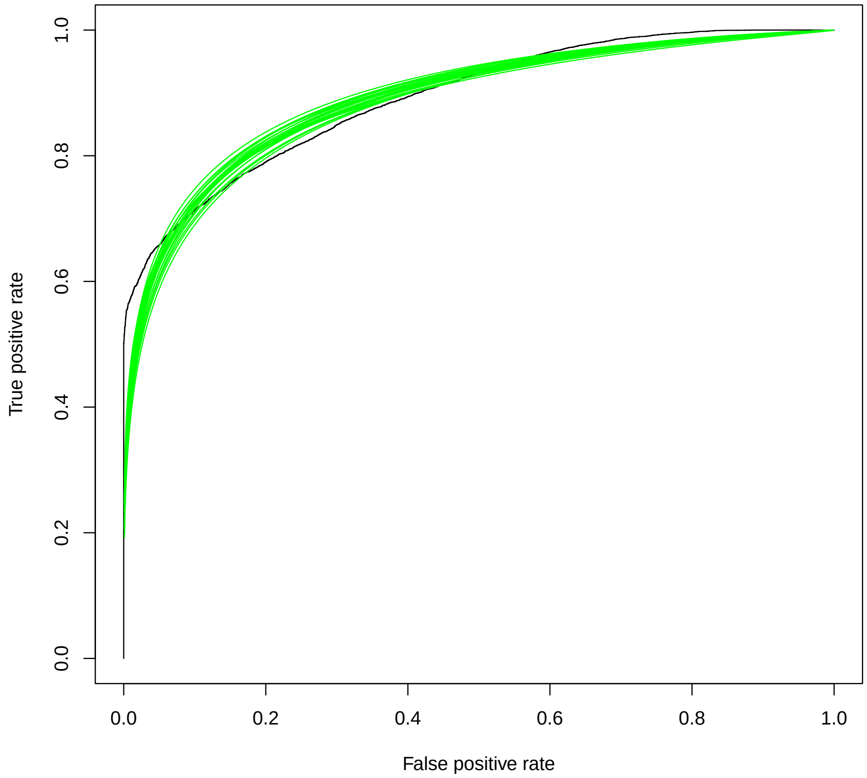

Figure S4: Empirical ROC curve vs. ROC-GLM (green) on 20 simulated data sets with score values from skewd distributions.

R Code for Figure ??

```
# Gamma distributed scores and bias of the ROC-GLM

n.0<-10000
x.0<-8-rgamma(n.0,shape=4)
n.1<-10000
x.1<-rgamma(n.1,shape=8)
mean(x.0)
mean(x.1)
boxplot(x.0,x.1)
pred.01<-c(x.0,x.1)
labe.01<-rep(c(0,1),c(n.0,n.1))
library(ROCR)
pred.obj<-prediction(pred.01,labe.01)
perf.obj<-performance(pred.obj, "tpr", "fpr")
plot(perf.obj); grid

# ROC-GLM

roc.glm.rfc<-function(marker=data.diag$marker,
                      class=data.diag$class)
{
  N.0<-sum(class==0)
  T.T<-(1:(N.0-1)/N.0)
  M.0<-marker[class==0]
  M.0<-M.0[order(M.0)]
  M.1<-marker[class==1]
  M.1<-M.1[order(M.1)]

  PW.0<-!outer(M.0,M.1,"<")

  neg.place.values<-apply(PW.0,2,mean)

  U.it<-!outer(neg.place.values,T.T,">")
  dd.1<-nrow(U.it)
  dd.2<-ncol(U.it)

  pseudo.data<-NULL
  for (i in 1:dd.1)
  {
    yy<-U.it[i,]
    xx<-qnorm(T.T)
    pseudo.data<-rbind(pseudo.data,cbind(xx,yy))
  }
  pseudo.data<-as.data.frame(pseudo.data)
  names(pseudo.data)<-c("xx","yy")
  glm.pseudo<-glm(yy~xx,family=binomial(link="probit"),
```

```
                    data=pseudo.data)

  cc<-coefficients(glm.pseudo)
  return(cc)
}
roc.glm.function.rfc<-function(x=0.2,cc=coef.roc.glm)
{
  return(pnorm(cc[1]+qnorm(x)*cc[2]))
}

n.simul<-20
for (k in 1:n.simul)
{
  m.0<-1000
  x.0<-8-rgamma(m.0,shape=4)
  m.1<-1000
  x.1<-rgamma(m.1,shape=8)
  pred.m<-c(x.0,x.1)
  labe.m<-rep(c(0,1),c(m.0,m.1))
  coef.roc.glm<-roc.glm.rfc(pred.m,labe.m)
  FPR.values<-seq(0.001,0.999,by=0.001)
  TPR.values<-roc.glm.function.rfc(FPR.values,)
  lines(FPR.values,TPR.values,col="green")
}
```
