# Supplementary figures and images for: Distributed non-disclosive validation of predictive models by a modified ROC-GLM
Source: BMC Med Res Methodol. 2024 Aug 29;24:190. doi: 10.1186/s12874-024-02312-4 (PMC11363434; doi:10.1186/s12874-024-02312-4)

# Gaussian Mechanism for $\Delta_2(f) = 0.01$

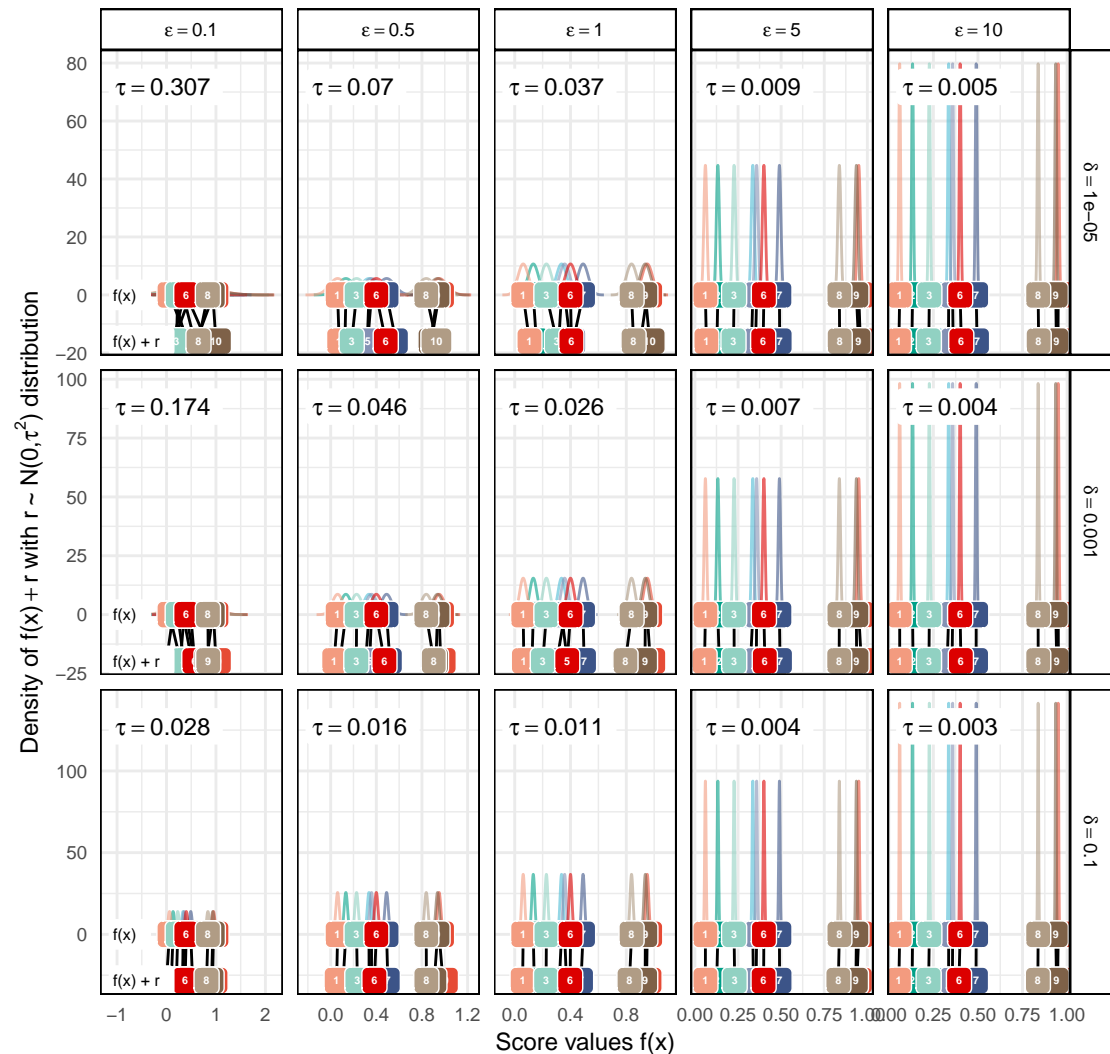

Supplement: Supplementary file 1 — Supplementary Material 1. [file 12874_2024_2312_MOESM1_ESM.zip › appendix/figures/gaussian-mechanism0.01.pdf]

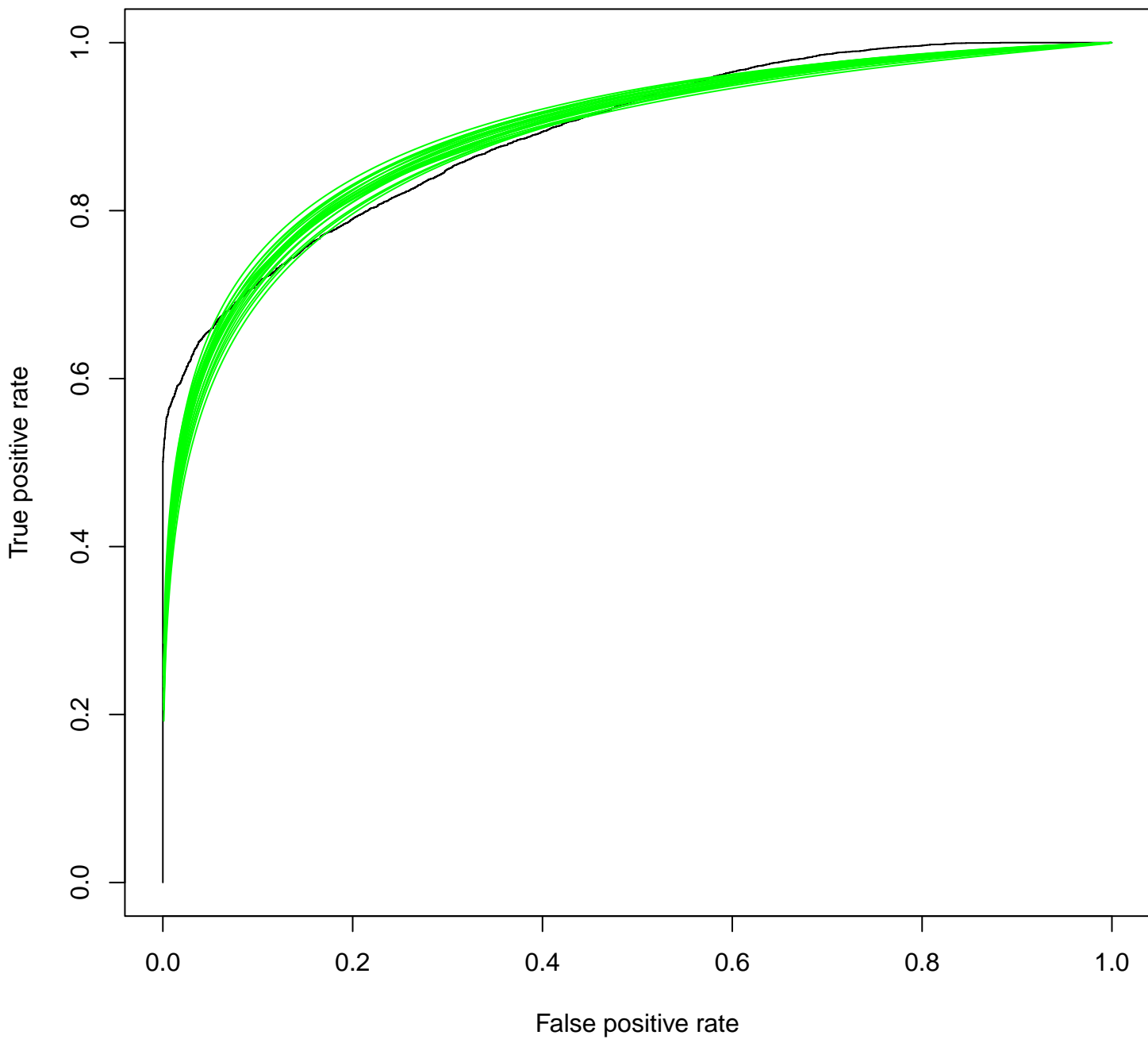

Supplement: Supplementary file 1 — Supplementary Material 1. [file 12874_2024_2312_MOESM1_ESM.zip › appendix/figures/ROC_vs_ROCGLM_gamma.pdf]

# Gaussian Mechanism for $\Delta_2(f) = 0.1$

Density of  $f(x) + r$  with  $r \sim N(0, \tau^2)$  distribution

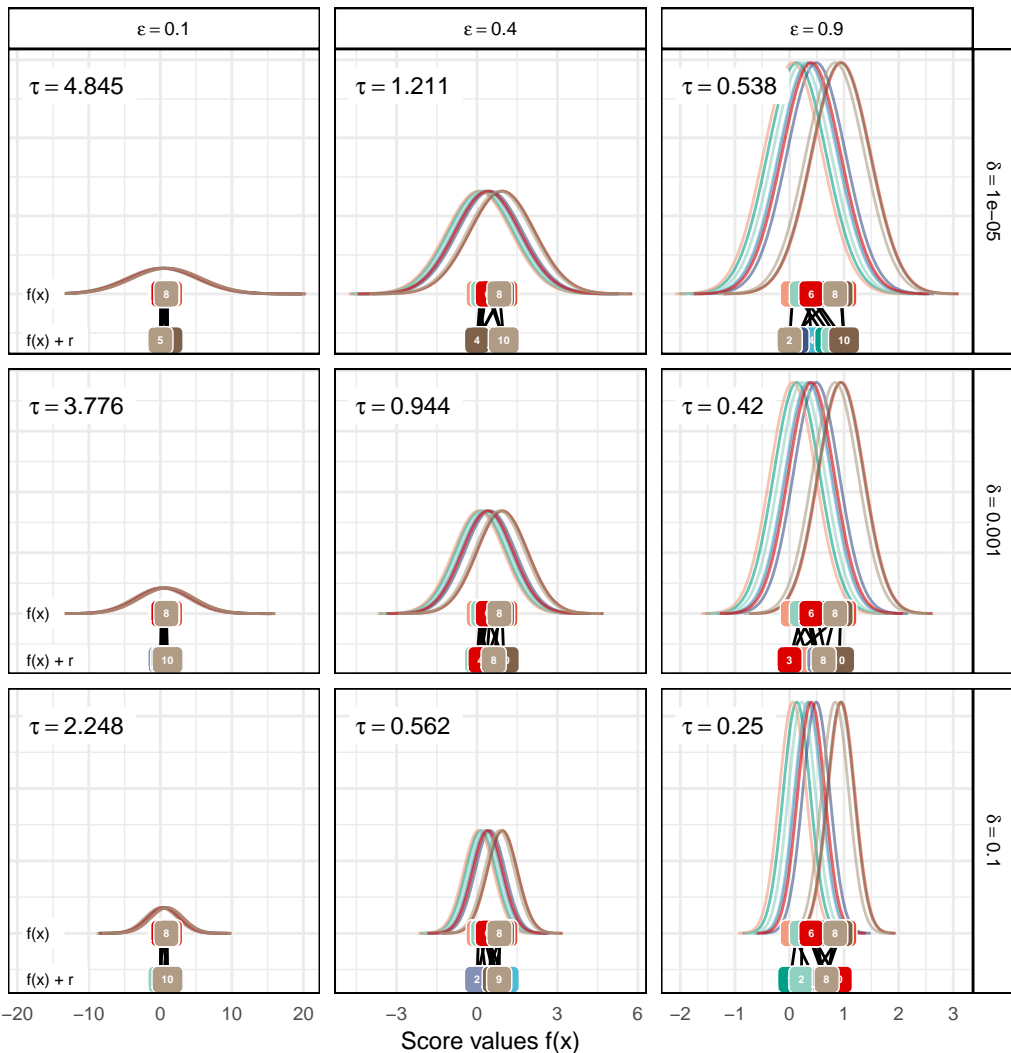

Supplement: Supplementary file 1 — Supplementary Material 1. [file 12874_2024_2312_MOESM1_ESM.zip › appendix/figures/gaussian-mechanism0.1.pdf]

# Gaussian Mechanism for $\Delta_2(f) = 0.2$

Density of  $f(x) + r$  with  $r \sim N(0, \tau^2)$  distribution

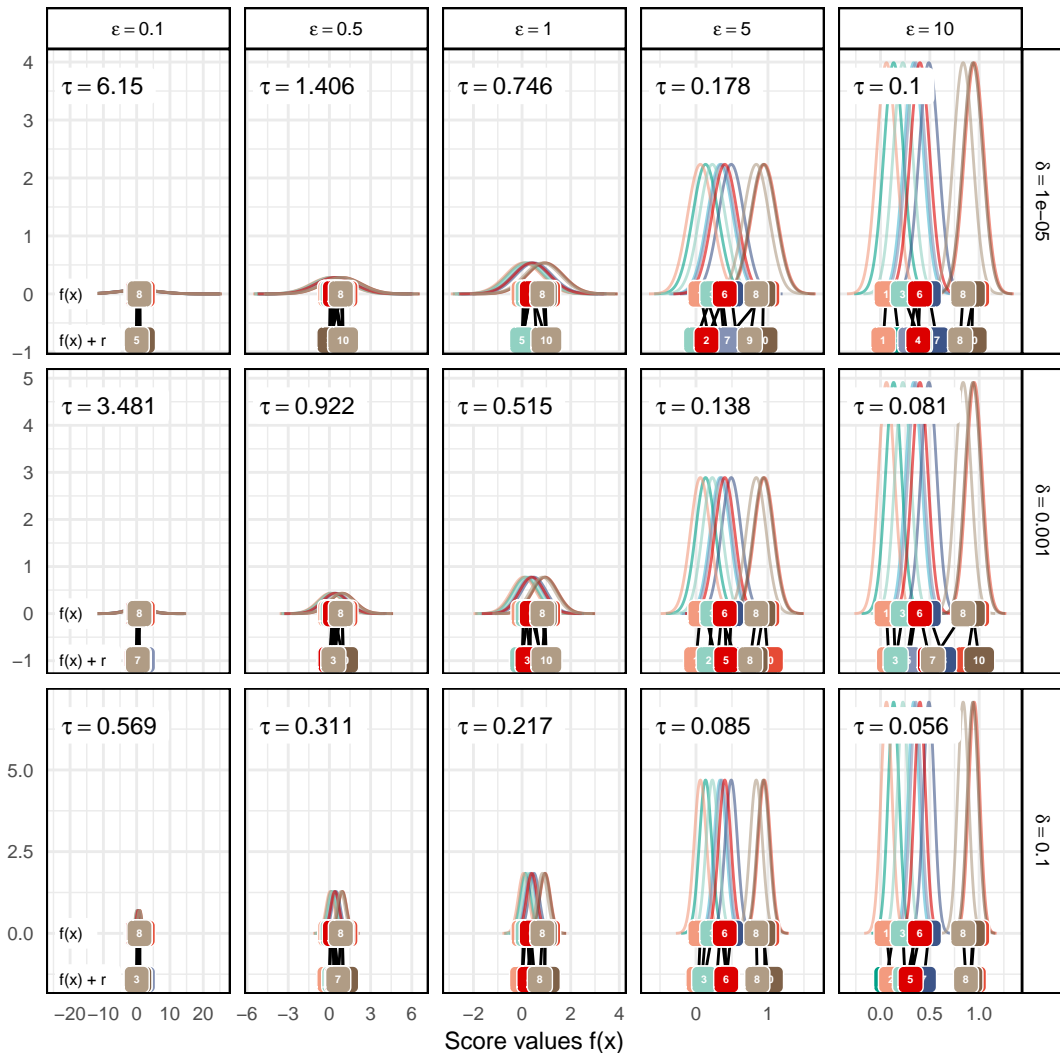

Supplement: Supplementary file 1 — Supplementary Material 1. [file 12874_2024_2312_MOESM1_ESM.zip › appendix/figures/gaussian-mechanism0.2.pdf]

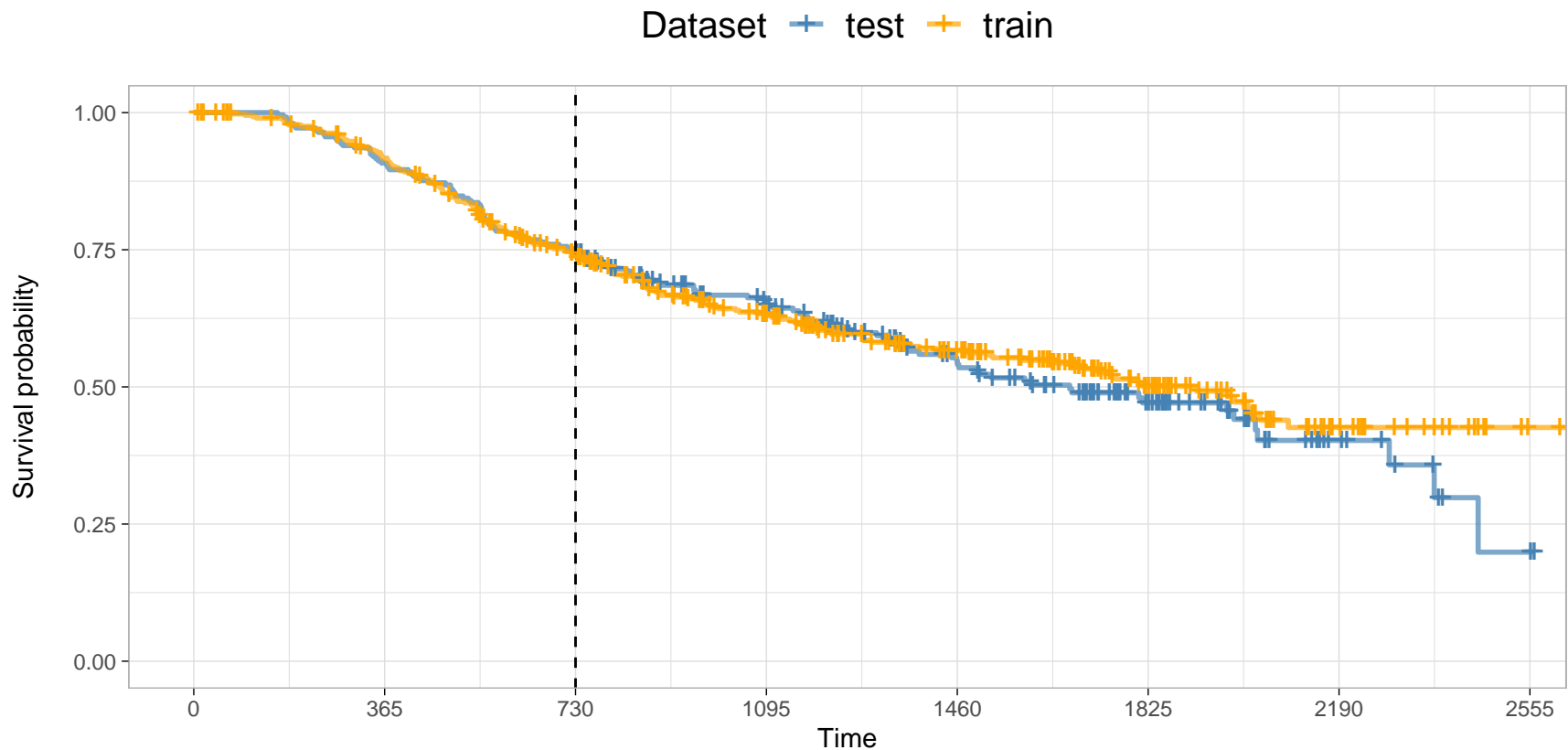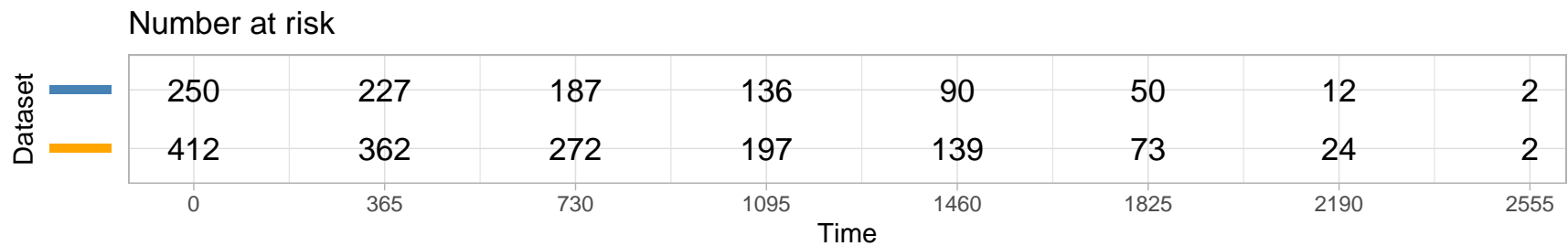

Supplement: Supplementary file 1 — Supplementary Material 1. [file 12874_2024_2312_MOESM1_ESM.zip › appendix/figures/survplot_GBSG2.pdf]

# Gaussian Mechanism for $\Delta_2(f) = 0.4$

Density of  $f(x) + r$  with  $r \sim N(0, \tau^2)$  distribution

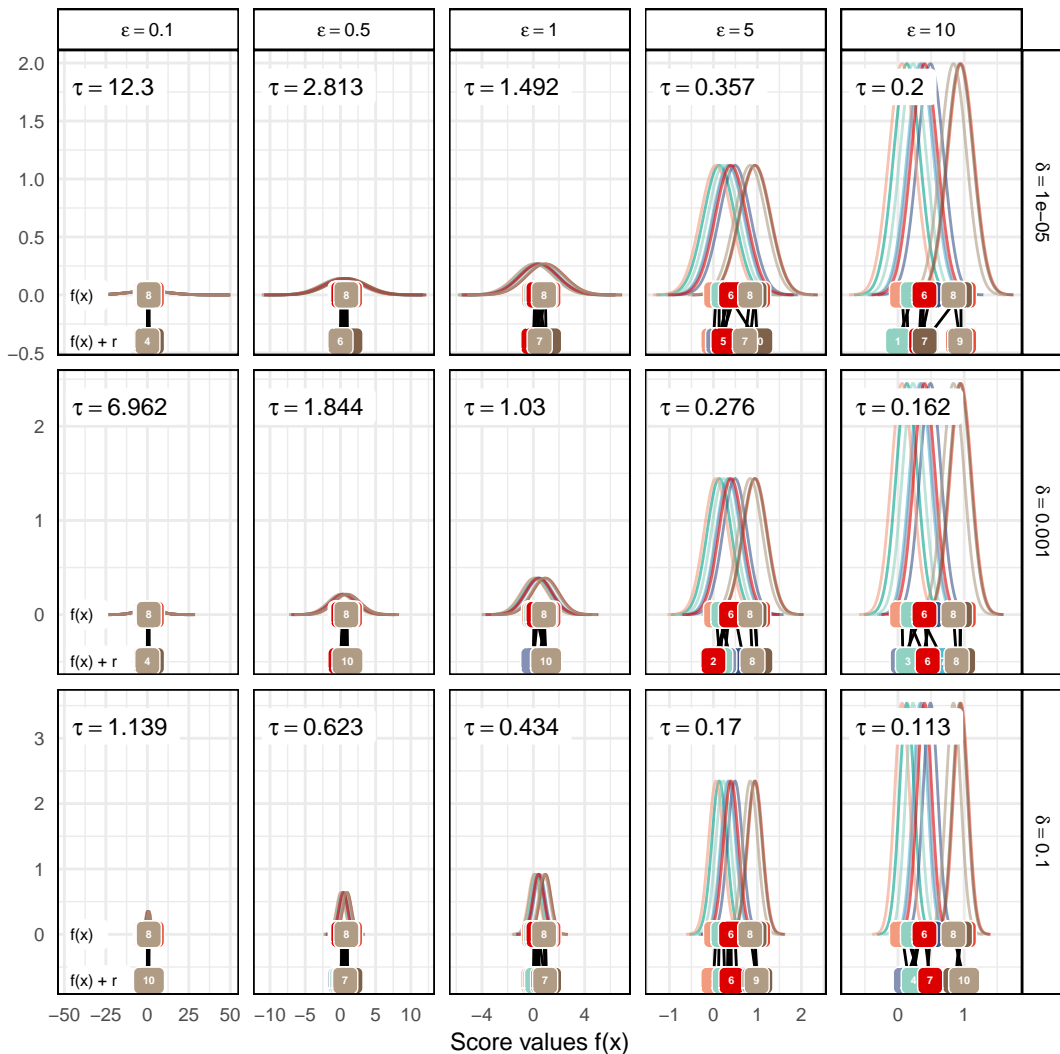

Supplement: Supplementary file 1 — Supplementary Material 1. [file 12874_2024_2312_MOESM1_ESM.zip › appendix/figures/gaussian-mechanism0.4.pdf]
